# The *LsVe1L* allele provides a molecular marker for resistance to *Verticillium dahliae* race 1 in lettuce

**DOI:** 10.1186/s12870-019-1905-9

**Published:** 2019-07-10

**Authors:** Patrik Inderbitzin, Marilena Christopoulou, Dean Lavelle, Sebastian Reyes-Chin-Wo, Richard W. Michelmore, Krishna V. Subbarao, Ivan Simko

**Affiliations:** 1 Department of Plant Pathology, University of California, Davis, CA 95616 USA; 2 Present address: Indigo Ag, Charlestown, MA 02129 USA; 3 Genome Center, University of California, Davis, CA 95616 USA; 4 Departments of Plant Sciences, Molecular & Cellular Biology, Medical Microbiology & Immunology, University of California, Davis, CA 95616 USA; 5 United States Department of Agriculture, Agricultural Research Service, Crop Improvement and Protection Research Unit, Salinas, CA 93905 USA

**Keywords:** *Lactuca sativa*, Genomics, Marker-assisted selection, Plant breeding, Wilt resistance

## Abstract

**Background:**

Verticillium wilt caused by the fungus *Verticillium dahliae* race 1 is among the top disease concerns for lettuce in the Salinas and Pajaro Valleys of coastal central California. Resistance of lettuce against *V. dahliae* race 1 was previously mapped to the single dominant *Verticillium resistance 1* (*Vr1*) locus. Lines of tomato resistant to race 1 are known to contain the closely linked *Ve1* and *Ve2* genes that encode receptor-like proteins with extracellular leucine-rich repeats; the Ve1 and Ve2 proteins act antagonistically to provide resistance against *V. dahliae* race 1. The *Vr1* locus in lettuce contains a cluster of several genes with sequence similarity to the tomato *Ve* genes*.* We used genome sequencing and/or PCR screening along with pathogenicity assays of 152 accessions of lettuce to investigate allelic diversity and its relationship to race 1 resistance in lettuce.

**Results:**

This approach identified a total of four *Ve* genes: *LsVe1*, *LsVe2*, *LsVe3*, and *LsVe4*. The majority of accessions, however, contained a combination of only three of these *LsVe* genes clustered on chromosomal linkage group 9 (within ~ 25 kb in the resistant cultivar La Brillante and within ~ 127 kb in the susceptible cultivar Salinas).

**Conclusions:**

A single allele, *LsVe1L,* was present in all resistant accessions and absent in all susceptible accessions. This allele can be used as a molecular marker for *V. dahliae* race 1 resistance in lettuce. A PCR assay for rapid detection of race 1 resistance in lettuce was designed based on nucleotide polymorphisms. Application of this assay allows identification of resistant genotypes in early stages of plant development or at seed-level without time- and labor-intensive testing in the field.

**Electronic supplementary material:**

The online version of this article (10.1186/s12870-019-1905-9) contains supplementary material, which is available to authorized users.

## Background

The Salinas and Pajaro Valleys of coastal central California are among the most important lettuce-producing regions in the United States [1]. One of the top disease concerns for lettuce in the area is Verticillium wilt caused by the fungus *Verticillium dahliae* [2, 3], which is a soilborne pathogen with a wide host range that also includes artichoke, cotton, eggplant, hops, potato, sunflower, tobacco, and tomato [4, 5]. Two races of *V. dahliae* occur in coastal central California based on their differential virulence on cultivar La Brillante [6]; however, race 1 is more prevalent and economically important than race 2 [7]. In tomato, race 1 of *V. dahliae* carries *Ave1* that is recognized by *Ve1* in resistant genotypes [8]. *Ve* genes encode receptor-like proteins (RLPs) with extracellular leucine-rich repeats [9, 10]; such RLPs are widespread in land plants [11]. In addition to *Ve1,* tomato also contains the closely linked paralog *Ve2*; their encoded RLPs work antagonistically to confer resistance to *V. dahliae* race 1 [12]. Several *Ve* paralogs also confer resistance in otherwise *V. dahliae-*susceptible species including cotton [13], potato [14, 15], hops, and wild eggplant [11], but it is unknown whether they function analogously to the tomato *Ve* genes in conferring *V. dahliae* race 1 resistance. In lettuce, resistance to *V. dahliae* race 1 was originally identified in the Batavia-type cultivar, La Brillante, as conferred by a single dominant locus (*Verticillium resistance 1*, *Vr1*) located on chromosomal linkage group 9 [16]. The lettuce *Vr1* locus contains several genes with sequence similarity to the *Ve* genes of tomato; it is very likely that one or more of these *LsVe* homologs are functional resistance genes.

The goals of this study were to identify the lettuce *Ve* allele(s) that play a role in resistance to *V. dahliae* race 1 and to develop PCR-based assays for marker-assisted selection. For this purpose, we analyzed the genome sequences of cultivars La Brillante (resistant to *V. dahliae* race 1) and the previously published Salinas [17] (iceberg type, susceptible to *V. dahliae* race 1). Subsequently, we sequenced and/or used allele-specific PCR screens of 150 additional lettuce accessions to identify the allele(s) of the *LsVe* genes that are exclusively present in resistant phenotypes.

## Results

### Phenotypic evaluation of resistance in field tests

One hundred and fifty accessions from ten horticultural types and *L. serriola* were evaluated in four field experiments. Twenty accessions (13.3%) showed no disease symptoms and were considered resistant. The proportion of disease incidence in susceptible accessions ranged from 0.07 to 1.00, with a mean disease incidence of 0.43 (± 0.02). There was a substantial difference in the distribution of resistant phenotypes across horticultural types. Among horticultural types with at least five tested accessions, the largest frequency of resistant accessions was found in Latin (6/7 = 85.7%), followed by Batavia (2/6 = 33.3%), red leaf (4/15 = 26.7%), and butterhead (3/14 = 21.4%; Table 1) types. In contrast, the lowest frequencies of resistant accessions were found in iceberg (0/46 = 0%), romaine (2/36 = 5.6%), and green leaf (2/18 = 11.1%) types. Oil (0/4 = 0%), stem (1/3 = 33.3%) types, and *L. serriola* (0/1 = 0%) had fewer than five tested accessions each. All oil type accessions were susceptible to the disease and had a very high disease incidence (0.98 in one accession, 1.00 in all others). Statistical analysis indicated that the frequency of resistant accessions was significantly (*p* < 0.01) higher than the overall frequency of 13.3% in Latin types, while it was significantly lower in iceberg types; however, the statistical power to detect significant differences for horticultural types with a small number of tested accessions is limited.

**Table 1:** Difference among horticultural types in their phenotypic reaction to *V. dahliae* race 1 tested in field trials

Horticultural type or species	No. of tested accessions	No. of accessions with disease incidence					Frequency of resistant accessions	Chi-square test ^a^
		0.00 (resistant)	0.01–0.25	0.26–0.50	0.51–0.75	0.76–1.00		
Batavia	6	2		4			0.33	1.6
Butterhead	14	3	9	1		1	0.21	0.8
Green leaf	18	2	2	10	2	2	0.11	0.1
Iceberg	46		10	23	10	3	0.00	12.8*
Latin	7	6	1				0.86	19.0*
Oil	4					4	0.00	1.1
Red leaf	15	4		3	4	4	0.27	2.0
Romaine	36	2	9	20	4	1	0.06	2.2
Stem	3	1		2			0.33	0.8
*Lactuca serriola*	1				1		0.00	0.3
Total	150	20	31	63	21	15	0.13	

^a^ Values of two-sided χ2 test for the frequency of resistant accessions. Asterisk (*) indicates horticultural types with the frequency of resistant accessions significantly different at experiment-wise *p* < 0.01 from the overall frequency (0.13) observed for all tested types. Note that the statistical test has a low to absent detection power for significant results for horticultural types with a very few tested accessions

### Lettuce genome assemblies

Genome assemblies were generated for 61 accessions of cultivated lettuce (Table 2). The assembly of cultivar La Brillante consisted of 41,939 scaffolds with a total length of 2.04 Gb and had an L_50_ of 90.84 kb. The remaining 60 draft de novo assemblies consisted of 1.0 to 3.2 M contigs (average 2.78 M) with a total length of 2.08 to 2.44 Gb (average 2.20 Gb) and an L_50_ of 1.22 to 3.66 kb (average 1.6 kb). Reads have been submitted to GenBank (BioProject PRJNA478460).

**Table 2:** Accessions used in this study sorted by horticultural type and accession name

Type	Accession	Identifier	Genome	Race 1 resistance	*LsVe1L*	*LsVe3L*	*LsVe4L*	*LsVe1S*	*LsVe2S*	*LsVe3S*	*No. of tested plants*	*Proportion of symptomatic plants* ^k^
Batavia	Anuenue^a, b^	L2	+	–	–	–	–	–	+	+	90	0.26 (0.18–0.35)
Batavia	Batavia Reine des Glaces^c, b^	L90	–	–	–	ND^i^	–	ND	ND	ND	90	0.44 (0.35–0.55)
Batavia	Iceberg^c, b^	L115	–	–	–	ND	–	ND	ND	ND	30	0.50 (0.33–0.67)
Batavia	La Brillante^d, e^	10G364–1	+	+	+	+	+	–	–	–	NT^j^	NT
Batavia	La Brillante^c, b^	L119	–	+	+	ND	+	ND	ND	ND	180	0.00 (0.00–0.02)
Batavia	Laura^d, b^	L43	+	+	+	+	+	–	–	–	60	0.00 (0.00–0.06)
Batavia	Reines des Glaces^d, b^	L53	+	–	–	–	–	+	+	+	30	0.40 (0.25–0.58)
Butterhead	Allegiance^a, b^	L85	–	–	–	ND	+	ND	ND	ND	30	0.17 (0.07–0.34)
Butterhead	Ancora^a, f^	L240	–	+	+	ND	+	ND	ND	ND	60	0.00 (0.00–0.06)
Butterhead	Bennett^a, b^	L93	–	–	–	ND	+	ND	ND	ND	30	0.20 (0.10–0.37)
Butterhead	Bibb^d, b^	L26	+	–	–	–	–	+	+	+	30	0.07 (0.02–0.21)
Butterhead	Cobham Green^a, b^	L6	+	–	–	+	+	+	+	–	30	0.80 (0.63–0.91)
Butterhead	Dark Green Boston-C^d, b^	L71	+	–	–	–	+	+	+	–	26	0.23 (0.11–0.42)
Butterhead	Grappa^a, b^	L106	–	–	–	ND	–	ND	ND	ND	30	0.07 (0.02–0.21)
Butterhead	Little Gem-G^d, b^	L77	+	+	+	+	+	–	–	–	60	0.00 (0.00–0.06)
Butterhead	Margarita^d, b^	L45	+	–	–	–	+	+	+	–	30	0.30 (0.17–0.48)
Butterhead	Mariska^d, b^	L46	+	–	–	–	+	+	+	–	29	0.24 (0.12–0.42)
Butterhead	Martin^a, b^	L124	–	–	–	ND	–	ND	ND	ND	30	0.17 (0.07–0.34)
Butterhead	Ostinata^a, b^	L127	–	–	–	ND	+	ND	ND	ND	30	0.07 (0.02–0.21)
Butterhead	Tania^a, b^	L149	–	–	–	ND	+	ND	ND	ND	30	0.07 (0.02–0.21)
Butterhead	Tinto^a, b^	L23	+	+	+	+	+	–	–	–	59	0.00 (0.00–0.06)
Green leaf	Alpine^a, b^	L86	–	–	–	ND	–	ND	ND	ND	30	0.40 (0.25–0.58)
Green leaf	Franklin^a, b^	L100	–	–	–	ND	–	ND	ND	ND	60	0.20 (0.12–0.32)
Green leaf	Genecorps Green^a, b^	L103	–	–	–	ND	–	ND	ND	ND	30	0.60 (0.42–0.75)
Green leaf	Grand Rapids^d, b^	L37	+	–	–	–	–	+	+	+	60	0.45 (0.33–0.58)
Green leaf	Green Vision^a, b^	L109	–	–	–	ND	–	ND	ND	ND	30	0.20 (0.10–0.37)
Green leaf	Hacienda^d, b^	L40	+	–	–	–	–	+	+	+	30	0.97 (0.83–0.99)
Green leaf	Plymouth^a, b^	L19	+	+	+	+	+	–	–	–	120	0.00 (0.00–0.03)
Green leaf	Pybas Green^a, b^	L133	–	–	–	ND	–	ND	ND	ND	30	0.37 (0.22–0.54)
Green leaf	Salad Bowl^a, f^	L307	–	–	–	ND	–	ND	ND	ND	27	0.41 (0.25–0.59)
Green leaf	Shining Star^a, b^	L141	–	–	–	ND	–	ND	ND	ND	30	0.50 (0.33–0.67)
Green leaf	Slobolt^d, b^	L56	+	–	–	–	–	+	+	+	29	0.45 (0.28–0.62)
Green leaf	Tehama^a, b^	L150	–	–	–	ND	–	ND	ND	ND	30	0.47 (0.30–0.64)
Green leaf	Tropicana^d, b^	L58	+	–	–	–	–	+	+	+	30	0.70 (0.52–0.83)
Green leaf	Two Star^a, b^	L25	+	–	–	–	–	+	+	+	150	0.39 (0.31–0.47)
Green leaf	Waldmann’s Green^a, b^	L160	–	–	–	ND	–	ND	ND	ND	30	0.50 (0.33–0.67)
Green leaf	Waldmans Green-G^d, b^	L84	+	–	–	–	–	+	+	+	30	0.37 (0.22–0.54)
Green leaf	Western Green^a, b^	L161	–	–	–	ND	–	ND	ND	ND	30	0.90 (0.74–0.97)
Green leaf	Xena^d, b^	L63	+	+	+	+	+	–	–	–	59	0.00 (0.00–0.06)
Iceberg	Autumn Gold^d, b^	L66	+	–	–	–	–	+	+	+	28	0.54 (0.36–0.70)
Iceberg	Bayview^a, b^	L92	–	–	–	ND	–	ND	ND	ND	30	0.30 (0.17–0.48)
Iceberg	Big Ben^a, b^	L94	–	–	–	ND	–	ND	ND	ND	30	0.27 (0.14–0.44)
Iceberg	Calicel^d, b^	L27	+	–	–	–	–	+	+	+	26	0.54 (0.35–0.71)
Iceberg	Calmar^a, b^	L5	+	–	–	–	–	+	+	+	30	0.50 (0.33–0.67)
Iceberg	Cannery Row^a, b^	L163	–	–	–	ND	–	ND	ND	ND	30	0.20 (0.10–0.37)
Iceberg	Cisco^d, b^	L30	+	–	–	–	–	+	+	+	26	0.65 (0.46–0.81)
Iceberg	Coolguard^d, b^	L31	+	–	–	–	–	+	+	+	28	0.86 (0.69–0.94)
Iceberg	Corona^a, b^	L169	–	–	–	ND	–	ND	ND	ND	30	0.20 (0.10–0.37)
Iceberg	Diplomat^d, b^	L34	+	–	–	–	–	+	+	+	29	0.55 (0.38–0.72)
Iceberg	Durango^a, b^	L173	–	–	–	ND	–	ND	ND	ND	30	0.30 (0.17–0.48)
Iceberg	Early Bird^a, b^	L35	+	–	–	–	–	+	+	+	30	0.40 (0.25–0.58)
Iceberg	El Dorado^a, b^	L174	–	–	–	ND	–	ND	ND	ND	30	0.20 (0.10–0.37)
Iceberg	Empire^a, b^	L175	–	–	–	ND	–	ND	ND	ND	30	0.30 (0.17–0.48)
Iceberg	Grand Slam^a, b^	L105	–	–	–	ND	–	ND	ND	ND	30	0.27 (0.14–0.44)
Iceberg	Great Lakes^a, b^	L107	–	–	–	ND	–	ND	ND	ND	30	0.40 (0.25–0.58)
Iceberg	Great Lakes 659-G^d, b^	L73	+	–	–	–	–	+	+	+	30	0.37 (0.22–0.54)
Iceberg	Hallmark^a, b^	L111	–	–	–	ND	–	ND	ND	ND	30	0.30 (0.17–0.48)
Iceberg	Home Run^a, b^	L182	–	–	–	ND	–	ND	ND	ND	30	0.37 (0.22–0.54)
Iceberg	Icon^a, b^	L116	–	–	–	ND	–	ND	ND	ND	30	0.30 (0.17–0.48)
Iceberg	Ithaca ZAA-C^d, b^	L74	+	–	–	–	–	+	+	+	30	0.83 (0.66–0.93)
Iceberg	Laguna Fresca^a, b^	L120	–	–	–	ND	–	ND	ND	ND	30	0.17 (0.07–0.34)
Iceberg	Legend^a, b^	L121	–	–	–	ND	–	ND	ND	ND	30	0.30 (0.17–0.48)
Iceberg	Liberty^a, b^	L122	–	–	–	ND	–	ND	ND	ND	30	0.37 (0.22–0.54)
Iceberg	Mesa 659-C^d, b^	L78	+	–	–	–	–	+	+	+	30	0.80 (0.63–0.91)
Iceberg	Monument^a, b^	L125	–	–	–	ND	–	ND	ND	ND	30	0.17 (0.07–0.34)
Iceberg	Pacific^a, b^	L128	–	–	–	ND	–	ND	ND	ND	60	0.18 (0.11–0.30)
Iceberg	Primus^a, b^	L20	+	–	–	–	–	+	+	+	30	0.57 (0.39–0.73)
Iceberg	Salinas^g^	NA^h^	NA	–	–	–	–	+	+	+	NT	NT
Iceberg	Salinas^c, b^	L138	–	–	–	ND	–	ND	ND	ND	180	0.55 (0.48–0.62)
Iceberg	Salinas 88^c, b^	L139	–	–	–	ND	–	ND	ND	ND	120	0.48 (0.40–0.57)
Iceberg	Salinas 88-G^d, b^	L81	+	–	–	–	–	+	+	+	30	0.40 (0.25–0.58)
Iceberg	Sharp Shooter^a, b^	L140	–	–	–	ND	–	ND	ND	ND	30	0.60 (0.42–0.75)
Iceberg	Silverado^a, b^	L143	–	–	–	ND	–	ND	ND	ND	30	0.30 (0.17–0.48)
Iceberg	Sniper^a, b^	L144	–	–	–	ND	–	ND	ND	ND	60	0.55 (0.42–0.67)
Iceberg	Sun Devil^a, b^	L146	–	–	–	ND	–	ND	ND	ND	30	0.20 (0.10–0.37)
Iceberg	Sure Shot^a, b^	L148	–	–	–	ND	–	ND	ND	ND	30	0.20 (0.10–0.37)
Iceberg	Telluride^a, b^	L151	–	–	–	ND	–	ND	ND	ND	90	0.34 (0.25–0.45)
Iceberg	Tiber^a, b^	L152	–	–	–	ND	–	ND	ND	ND	60	0.53 (0.41–0.65)
Iceberg	Tribute^a, b^	L153	–	–	–	ND	–	ND	ND	ND	30	0.40 (0.25–0.58)
Iceberg	Trojan^a, b^	L155	–	–	–	ND	–	ND	ND	ND	30	0.30 (0.17–0.48)
Iceberg	Vandenberg^a, b^	L156	–	–	–	ND	–	ND	ND	ND	30	0.47 (0.30–0.64)
Iceberg	Vanguard^a, b^	L157	–	–	–	ND	–	ND	ND	ND	30	0.20 (0.10–0.37)
Iceberg	Vanguard-C^d, b^	L82	+	–	–	–	–	+	+	+	30	0.30 (0.17–0.48)
Iceberg	Vanguard-G^d, b^	L83	+	–	–	–	–	+	+	+	30	0.23 (0.12–0.41)
Iceberg	Venus^a, b^	L183	–	–	–	ND	–	ND	ND	ND	30	0.47 (0.30–0.64)
Iceberg	Winterhaven^d, b^	L61	+	–	–	–	–	+	+	+	23	0.61 (0.41–0.78)
Latin	Barnwood Gem^a, b^	L89	–	+	+	ND	+	ND	ND	ND	60	0.00 (0.00–0.06)
Latin	Brigade^a, b^	L97	–	–	–	ND	–	ND	ND	ND	30	0.17 (0.07–0.34)
Latin	Gallega^a, b^	L102	–	+	+	ND	+	ND	ND	ND	60	0.00 (0.00–0.06)
Latin	Eruption^c, b^	L9	+	+	+	+	+	–	–	–	120	0.00 (0.00–0.03)
Latin	Little Gem^c, b^	L123	–	+	+	ND	+	ND	ND	ND	60	0.00 (0.00–0.06)
Latin	Little Gem^d, b^	L44	+	+	+	+	+	–	–	–	60	0.00 (0.00–0.06)
Latin	Pavane^a, b^	L16	+	+	+	+	+	–	–	–	90	0.00 (0.00–0.04)
Oil	PI 250020^a, b^	L17	+	–	–	–	–	+	+	+	30	1.00 (0.89–1.00)
Oil	PI 251245^d, b^	L50	+	–	–	–	–	+	+	+	30	1.00 (0.89–1.00)
Oil	PI 251245^a, b^	L18	–	–	–	ND	–	ND	ND	ND	30	1.00 (0.89–1.00)
Oil	PI 251246^c, b^	L131	–	–	–	ND	–	ND	ND	ND	60	0.98 (0.91–1.00)
Red leaf	Aragon Red^a, b^	L87	–	–	–	ND	–	ND	ND	ND	30	0.47 (0.30–0.64)
Red leaf	Battalion^a, b^	L91	–	+	+	ND	+	ND	ND	ND	60	0.00 (0.00–0.06)
Red leaf	Big Red^a, b^	L95	–	–	–	ND	–	ND	ND	ND	30	1.00 (0.89–1.00)
Red leaf	Deep Red^d, b^	L32	+	–	–	–	–	+	+	+	30	0.80 (0.63–0.91)
Red leaf	Lolla Rossa^a, b^	L14	+	+	+	+	+	–	–	–	60	0.00 (0.00–0.06)
Red leaf	Merlot^a, b^	L15	+	+	+	+	+	–	–	–	90	0.00 (0.00–0.04)
Red leaf	New Red^a, b^	L126	–	–	–	ND	–	ND	ND	ND	30	0.97 (0.83–0.99)
Red leaf	Prizehead^a, b^	L132	–	–	–	ND	–	ND	ND	ND	30	0.70 (0.52–0.83)
Red leaf	Red Fox^d, b^	L51	+	–	–	–	–	+	+	+	30	0.70 (0.52–0.83)
Red leaf	Red Grenoble^a, b^	L134	–	–	–	ND	+	ND	ND	ND	60	0.30 (0.20–0.43)
Red leaf	Red Rage^a, b^	L135	–	–	–	ND	–	ND	ND	ND	30	0.97 (0.83–0.99)
Red leaf	Red Tide^a, b^	L136	–	–	–	ND	–	ND	ND	ND	30	0.57 (0.39–0.73)
Red leaf	Red Tide^d, b^	L52	+	–	–	–	–	+	+	+	30	0.43 (0.27–0.61)
Red leaf	Sentry^a, b^	L21	+	+	+	+	+	–	–	–	180	0.00 (0.00–0.02)
Red leaf	Western Red Leaf^d, b^	L60	+	–	–	–	–	+	+	+	30	0.53 (0.36–0.70)
Romaine	Annapolis^c, b^	L1	+	+	+	+	+	–	–	–	90	0.00 (0.00–0.04)
Romaine	Avalanche^c, b^	L88	–	–	–	ND	–	ND	ND	ND	90	0.22 (0.15–0.32)
Romaine	Blonde Lente a Monter^c, b^	L4	+	–	–	–	–	+	+	+	30	0.70 (0.52–0.83)
Romaine	Brave Heart^c, b^	L96	–	–	–	ND	–	ND	ND	ND	60	0.32 (0.21–0.44)
Romaine	Caesar^c, b^	L98	–	–	–	ND	–	ND	ND	ND	30	0.17 (0.07–0.34)
Romaine	Camino Real^c, b^	L162	–	–	–	ND	–	ND	ND	ND	30	0.37 (0.22–0.54)
Romaine	Clemente^c, b^	L166	–	–	–	ND	–	ND	ND	ND	30	0.20 (0.10–0.37)
Romaine	Coastal Star^c, b^	L167	–	–	–	ND	–	ND	ND	ND	30	0.50 (0.33–0.67)
Romaine	Conquistador^c, b^	L168	–	–	–	ND	–	ND	ND	ND	30	0.27 (0.14–0.44)
Romaine	Costa Rica #4^d, b^	L70	+	–	–	–	–	+	+	+	30	0.43 (0.27–0.61)
Romaine	Costa Rica #4^c, b^	L170	–	–	–	ND	–	ND	ND	ND	30	0.33 (0.19–0.51)
Romaine	Darkland EL^c, b^	L171	–	–	–	ND	–	ND	ND	ND	30	0.37 (0.22–0.54)
Romaine	Defender^c, b^	L8	+	+	+	+	+	–	–	–	90	0.00 (0.00–0.04)
Romaine	EXP1752^a, b^	L99	–	–	–	ND	–	ND	ND	ND	30	0.17 (0.07–0.34)
Romaine	Flashy Troutback^c, b^	L10	+	–	–	+	+	–	+	–	30	0.30 (0.17–0.48)
Romaine	Fresh Heart^c, b^	L101	–	–	–	ND	–	ND	ND	ND	30	0.37 (0.22–0.54)
Romaine	Green Forest^c, f^	L189	–	–	–	ND	–	ND	ND	ND	28	0.46 (0.30–0.64)
Romaine	Green Towers^c, b^	L108	–	–	–	ND	–	ND	ND	ND	60	0.20 (0.10–0.37)
Romaine	Green Towers^d, b^	L39	+	–	–	–	–	+	+	+	30	0.23 (0.12–0.41)
Romaine	Hearts Delight^c, b^	L112	–	–	–	ND	–	ND	ND	ND	150	0.31 (0.24–0.38)
Romaine	Heavy Heart^c, b^	L113	–	–	–	ND	–	ND	ND	ND	30	0.30 (0.17–0.48)
Romaine	King Henry^c, b^	L117	–	–	–	ND	–	ND	ND	ND	30	0.20 (0.10–0.37)
Romaine	King Louie 2005^d, b^	L76	+	–	–	–	–	+	+	+	30	0.83 (0.66–0.93)
Romaine	Klamath^a, b^	L118	–	–	–	ND	–	ND	ND	ND	30	0.17 (0.07–0.34)
Romaine	Lee Tal^c, b^	L12	+	–	–	–	+	+	+	–	59	0.17 (0.09–0.28)
Romaine	Lobjoits Cos^c, b^	L13	+	–	–	–	–	+	+	+	90	0.53 (0.43–0.63)
Romaine	Parris Island Cos^c, b^	L129	–	–	–	ND	–	ND	ND	ND	60	0.45 (0.33–0.58)
Romaine	Parris Island Cos 714 (PIC714)^a, b^	L130	–	–	–	ND	–	ND	ND	ND	30	0.40 (0.25–0.58)
Romaine	Parris Island Cos-G^d, b^	L80	–	–	–	ND	–	ND	ND	ND	30	0.43 (0.27–0.61)
Romaine	Passport^d, b^	L64	+	–	–	–	–	+	+	+	30	0.50 (0.33–0.67)
Romaine	PI 171674^d, b^	L49	+	–	–	–	–	+	+	+	90	0.32 (0.23–0.42)
Romaine	SM09A^c, b^	L22	+	–	–	–	–	+	+	+	30	0.60 (0.42–0.75)
Romaine	Sunbelt^c, b^	L184	–	–	–	ND	–	ND	ND	ND	30	0.40 (0.25–0.58)
Romaine	Triple Threat^c, b^	L24	+	–	–	–	–	+	+	+	60	0.58 (0.46–0.70)
Romaine	Triton^c, b^	L154	–	–	–	ND	–	ND	ND	ND	30	0.47 (0.30–0.64)
Romaine	VJO3R^a, b^	L159	–	–	–	ND	–	ND	ND	ND	30	0.27 (0.14–0.44)
Stem	Balady Banha^c, b^	L3	+	+	+	+	+	–	–	–	88	0.00 (0.00–0.04)
Stem	Celtuce^a, b^	L165	–	–	–	ND	–	ND	ND	ND	30	0.40 (0.25–0.58)
Stem	Celtuce-G^d, b^	L69	+	–	–	–	–	+	+	+	30	0.43 (0.27–0.61)
*Lactuca serriola*	11-G99^c, f^	L185	–	–	–	ND	+	ND	ND	ND	30	0.70 (0.52–0.83)

^a^Germplasm Collection of USDA-ARS Salinas, CA

^b^Seeds

^c^Genome Wide Association Mapping Collection (GBS) of USDA-ARS Salinas, CA

^d^UC Davis Collection

^e^Dark-grown seedlings

^f^Leaf tissue from field

^g^Reyes-Chin-Wo et al., 2017

^h^Not applicable (NA), because this genome was sequenced previously (Reyes-Chin-Wo et al., 2017)

^i^Not determined (ND)

^j^Not field tested in the present study (NT). Cultivars La Brillante and Salinas we previously confirmed to be resistant and susceptible, respectively, to *V. dahliae* race 1 (Hayes at al., 2011)

^k^95% confidence interval for the proportion of symptomatic plants is shown in parentheses

### Ve genes and alleles of cultivars La Brillante, Salinas, and 60 other accessions

**Fig. 1:**
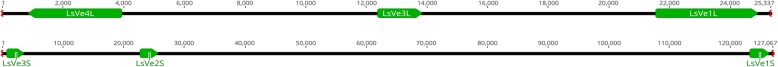
Partial scaffolds of lettuce cultivars La Brillante (Lsat_LaBrillante_v1_g_2266) and Salinas (lg_9) containing *LsVe* genes

**Fig. 2:**
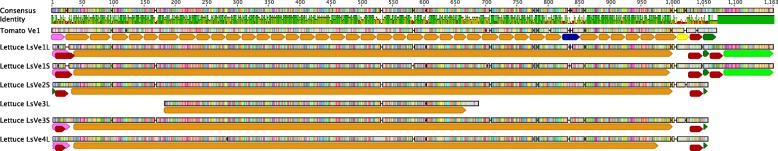
Alignment of tomato *Ve1* and *LsVe1, LsVe2, LsVe3*, and *LsVe4* alleles. Alleles are aligned in N to C orientation, residues are numbered across the top. Consensus and conserved residues across alleles are indicated in the top two tracks. Horizontal black boxes represent alleles, gray vertical lines represent substitutions, remaining colors inside black boxes represent different residues. Domains are indicated underneath alleles as colored boxes; the colors indicate the following: blue, non-LRR island domain (C2 domain); dark green, cytoplasmic domain; light green, non-cytoplasmic domain; orange, leucine-rich repeat region (individual repeats are indicated only for *Ve1*); pink, signal peptide; red, transmembrane domain; yellow, acidic domain. Domains follow [18] for *Ve1* (GenBank accession ACR33105)

**Fig. 3:**
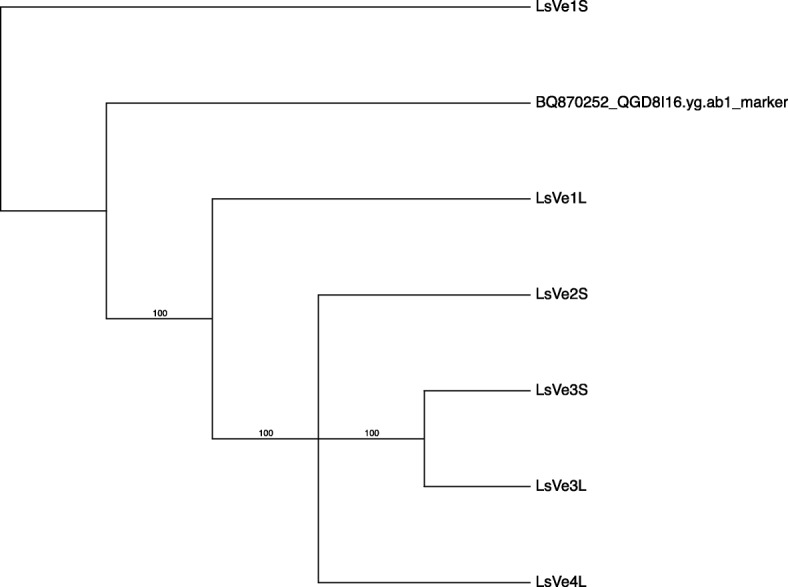
Unrooted parsimony bootstrap tree of cultivars La Brillante and Salinas *Ve* alleles. The topology shows that *LsVe1* alleles plus BQ870252_QGD8I16.yg.ab1 marker (GenBank accession BQ870252) [16] group together with maximum statistical support; the *LsVe3* alleles group together but *LsVe4L* and *LsVe2S* alleles do not. Bootstrap supports above 70% are indicated by the branches

The expressed sequence tag marker QGD8I16.yg.ab1 at the *Verticillium resistance 1* (*Vr1*) locus in lettuce [15] was used to query the genome assemblies of the lettuce cultivars La Brillante and Salinas using BLASTn. Three hits (e = 0.0) to scaffold linkage group 9 of the v8 reference assembly of the cultivar Salinas corresponded to three open reading frames (ORFs) that were named *LsVe1S* (because it had the highest sequence similarity of the three paralogs to *Ve1* of tomato), *LsVe2S*, and *LsVe3S* (Fig. 1). The encoded proteins were comprised of 1133 aa, 1041 aa, and 1039 aa for *LsVe1S, LsVe2S*, and *LsVe3S*, respectively, and including a signal peptide, 37 extracellular leucine-rich repeats, a transmembrane domain, and a cytoplasmic region inferred from the N- to C-terminus (Fig. 2, Additional file 1). *LsVe1S* had an additional potential transmembrane domain and non-cytoplasmic region. Similarly, there were three hits (e = 0.0) on contig Lsat_LaBrillante_v1_g_2266 for La Brillante. The hits corresponded to three gene models that differed in sequence from the three *Ve* genes in cultivar Salinas. Phylogenetic analyses showed that two of the ORFs grouped with maximum support with the *Ve1* and *Ve3* alleles in Salinas (Fig. 3) and were therefore named *LsVe1L* and *LsVe3L*, respectively. The third gene sequence was sufficiently different from all three genes in cultivar Salinas and was therefore named *LsVe4L* (Fig. 3). *LsVe1L, LsVe3L*, and *LsVe4L* encode proteins measuring 1136, 503, and 1043 aa*,* respectively. The domains encoded by *LsVe1L* and *LsVe4L* were the same as for *LsVe1S* and *LsVe3S,* respectively; however, while the sequence of *LsVe3L* is similar to *LsVe3S*, premature stop codons result in a truncated protein encoded by *LsVe3L* (Fig. 2, Additional file 1).

**Fig. 4:**
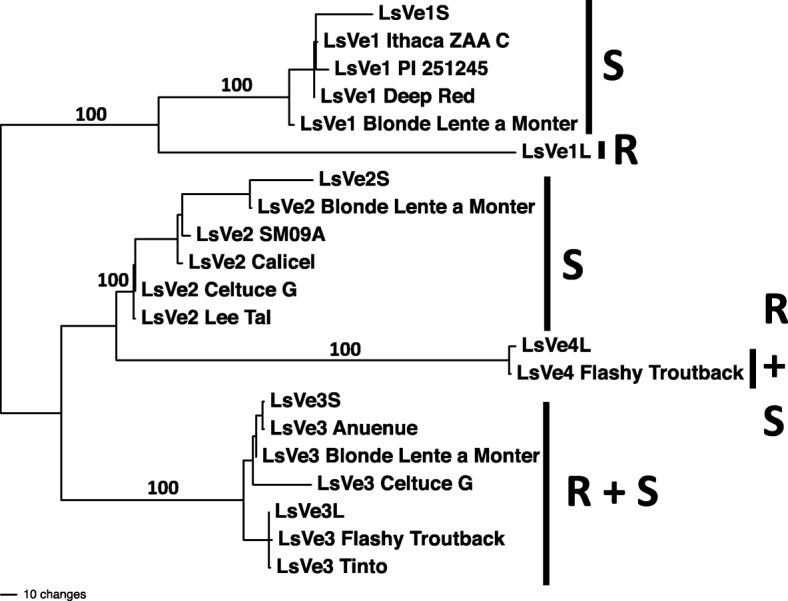
Phylogenetic tree of all *LsVe* alleles found in this study. Only one representative of each allele is included in the tree. Shown is one of 322 most parsimonious trees measuring 1120 steps; the tree is midpoint rooted. Taxon names consist of gene name followed by accession, except for cultivars La Brillante (L) and Salinas (S), where allele names are given. Numbers by the branches are bootstrap supports above 70%. Branch lengths are proportional to the number of changes occurring along the branches, the scale is given at the bottom. Association of alleles with resistance or susceptibility is indicated on the right side of vertical bars with R and S, respectively. All alleles are shown in Additional file 2

The La Brillante and Salinas *Ve* alleles were then used as queries to identify homologs in diverse germplasm of cultivated lettuce. A total of 180 *Ve* sequences were extracted from genome assemblies of 60 lettuce cultivars (Additional file 2). The sequences represented 21 different alleles that were identical or similar to the *Ve* alleles from La Brillante and Salinas (Fig. 4). The *LsVe1L* clade contained a single allele and the remaining clades contained between two and six alleles (Fig. 4, Additional file 2). This analysis likely underestimated the total number of *Ve* alleles because only 47 of the 186 *Ve* sequences included in this study represented complete genes (Additional file 2). All cultivars contained three *Ve* genes, except cultivar Anuenue (susceptible), in which only two alleles were detected that clustered in the *LsVe2* and *LsVe3* clades, and cultivar Cobham Green (susceptible) that contained four *Ve* genes that clustered in the *LsVs1S*, *LsVe2S*, *LsVe3L*, and *LsVe4L* clades. For the remaining *LsVe* genotypes, see Table 2.

There were substantial differences in frequencies of *LsVe* alleles among lettuce horticultural types. For example, all tested Iceberg cultivars had the identical combination of three alleles, *LsVe1S*, *LsVe2S*, and *LsVe3S*, while none of the genotyped Latin accessions contained any of these alleles (Table 3). Only six combinations of *LsVe* alleles were detected in 62 accessions with sequenced genomes. The five combinations were found in susceptible accessions: 40 accessions with *LsVe1S*, *LsVe2S*, and *LsVe3S*; four accessions with *LsVe4L*, *LsVe1S*, and *LsVe2S*; one accession with *LsVe3L*, *LsVe4L*, and *LsVe2S*; one accession with *LsVe3L*, *LsVe4L*, *LsVe1S*, and *LsVe2S*; and one accession with *LsVe2S* and *LsVe3S*. In addition, one combination of alleles was found in all (15) resistant accessions *LsVe1L*, *LsVe3L*, and *LsVe4L* (Table 3).

**Table 3:** *LsVe* allele frequencies in lettuce horticultural types included in this study. The number of accessions examined for the presence of the particular allele is given in parentheses

Type	*LsVe1L*	*LsVe3L*	*LsVe4L*	*LsVe1S*	*LsVe2S*	*LsVe3S*
Batavia	0.43 (7)	0.50 (4)	0.43 (7)	0.25 (4)	0.50 (4)	0.50 (4)
Butterhead	0.21 (14)	0.43 (7)	0.79 (14)	0.71 (7)	0.71 (7)	0.14 (7)
Green leaf	0.11 (18)	0.25 (8)	0.11 (18)	0.75 (8)	0.75 (8)	0.75 (8)
Iceberg	0 (47)	0 (16)	0 (47)	1.00 (16)	1.00 (16)	1.00 (16)
Latin	0.86 (7)	1.00 (3)	0.86 (7)	0 (3)	0 (3)	0 (3)
Oil	0 (4)	0 (2)	0 (4)	1.00 (2)	1.00 (2)	1.00 (2)
Red leaf	0.27 (15)	0.43 (7)	0.33 (15)	0.57 (7)	0.57 (7)	0.57 (7)
Romaine	0.06 (36)	0.23 (13)	0.11 (36)	0.77 (13)	0.85 (13)	0.69 (13)
Stem	0.33 (3)	0.50 (2)	0.33 (3)	0.50 (2)	0.50 (2)	0.50 (2)
*Lactuca serriola*	0 (1)	ND^a^	1.00 (1)	ND	ND	ND

^a^Not determined

Phylogenetic analyses of *Ve-*encoded amino acid sequences from cultivars La Brillante and Salinas with tomato *Ve1* and homologs from other Asteraceae*,* Cannabaceae*,* Malvaceae*,* and Solanaceae species showed that these lettuce *Ve* alleles were monophyletic with 99% bootstrap support. Two equally parsimonious trees were obtained and the tree length was 3492 steps (Additional file 3).

### PCR- based screening for LsVe1L and LsVe4L in 90 additional accessions

In order to determine the prevalence of candidate resistance alleles, 90 additional accessions were screened for the presence of *LsVe1L* and *LsVe4L* using allele-specific PCR (Additional file 4 and Additional file 5)*. LsVe1L*-specific products were detected in six accessions and *LsVe4L*-specific products in 12 accessions. All accessions with *LsVe1L* also had *LsVe4L* (Table 2)*. LsVe3L* screening was not performed because of the premature stop codons as mentioned above (Fig. 2).

### Diagnostic PCR assays for race 1 resistance based on LsVe1L

**Fig. 5:**
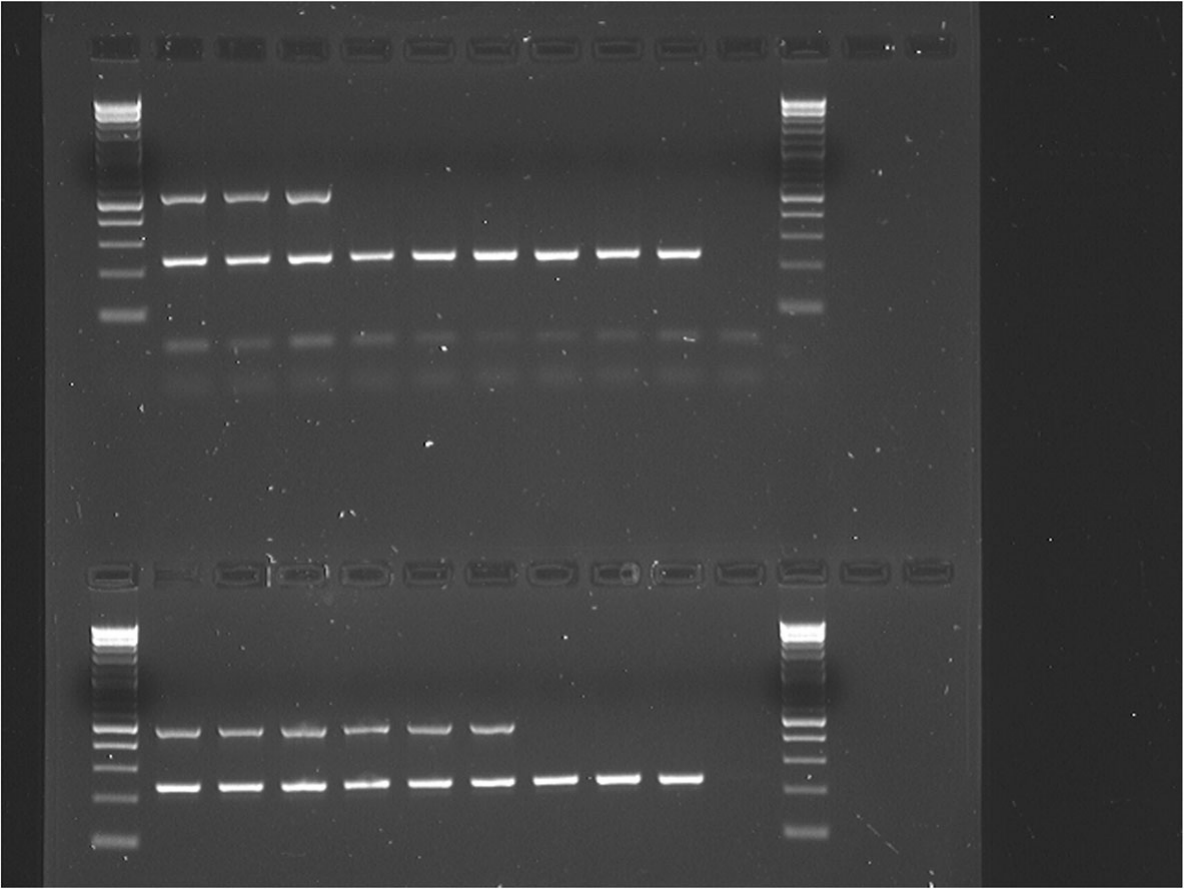
*LsVe1L* specific PCR assay is allele-specific. Shown are results of *LsVe1L-*specific PCR assays with selected lettuce accessions with known *LsVe* genotypes and resistance phenotypes. Resistance (R) and susceptibility (S) is indicated by capital letters for each accession. In all cases, the outcomes of the PCR assays were as expected from genome sequencing. Amplicon sizes are indicated by > and correspond to 200 and 500 bp. Lane numbers are: 1. 2-log ladder, 2. cultivar Balady Banha (*Ve* genotype: *LBVe1, LBVe4, LBVe3*), 3. cultivar Lolla Rossa (*LBVe1, LBVe4, LBVe3*), 4. cultivar Plymouth (*LBVe1, LBVe4, LBVe3*), 5. cultivar Cobham Green (*LBVe4, LBVe3, SVe1, SVe4*), 6. cultivar Lee Tal (*LBVe4, SVe1, SVe2*), 7. cultivar Margarita (*LBVe4, SVe1, SVe2*), 8. cultivar Anuenue (*SVe2, SVe3*), 9. cultivar Blonde Lente a Monter (*SVe1, SVe2, SVe3*), 10. cultivar Primus (*SVe1, SVe2, SVe3*), 11. negative control, and 12. 2-log ladder. PCR conditions are described in Table 4

*LsVe1L* and *LsVe1S* only share 89.5% sequence similarity differing by 358 single nucleotide polymorphisms (SNPs) and two indels. The overall ratio of non-synonymous (*d*N = 0.0754) to synonymous (*d*S = 0.2324) substitutions between the two alleles was 0.3246, providing no evidence for diversifying selection. These SNPs and indels provide multiple possibilities for allele-specific PCR-based assays. A PCR assay that selectively amplified *LsVe1L* was developed and validated as a marker for resistance to race 1 using selected accessions of lettuce with known *Ve* genotypes and resistance phenotypes. All PCR results were consistent with phenotypic observations and genome sequence data (Fig. 5). The *LsVe1L* allele was detected in 21 of the 152 tested accessions and all 21 were resistant to *V. dahliae* race 1 in field experiments (Table 2). Wilt symptoms were not observed on any of the 21 accessions with the exception of cultivar Plymouth, where two out of 30 plants showed root discoloration. However, the pathogen isolated from tap root tissue of cultivar Plymouth lacked the *V. dahliae* race 1 determinant *Ave1* [8], thus revealing that the symptoms were not caused by *V. dahliae* race 1 (Additional file 6). *LsVe4L* was present in all resistant but also some susceptible accessions (Table 2). This is consistent with *LsVe1L* rather than *LsVe4L* conferring resistance to *V. dahliae* race 1.

## Discussion

We tested 149 accessions of cultivated lettuce and a single accession of *L. serriola* in field experiments. Horticultural types with the greatest number of tested accessions were iceberg (46) and romaine (36) because they are the predominant types grown in the U.S. [1]. Despite the largest number of tested accessions, none of the iceberg cultivars were resistant to Verticillium wilt. This observation complements results from a previous study that tested accessions from multiple horticultural types for resistance to *V. dahliae* race 1 [19]. Therefore, development of modern iceberg-type cultivars with resistance to *V. dahliae* race 1 is one of the top priorities for public and private breeding efforts. USDA-ARS in Salinas released iceberg breeding lines [20–22] with their resistance derived from cultivar La Brillante. Cultivar La Brillante is a Batavia type lettuce with a small, round head that is less dense than those of modern iceberg cultivars. Because of the certain phenotypic similarities in the shape of heads, fewer backcrosses are usually needed to develop true to type iceberg cultivars when introgressing desirable genes from Batavia accessions than would be needed if those genes were introgressed from non-heading types of lettuces. Our current analyses showed that besides cultivar La Brillante, another Batavia cultivar (cultivar Laura) can also be used for a relatively rapid development of iceberg cultivars with resistance to *V. dahliae* race 1. Both of these cultivars contain the same combination of LsVe alleles (*LsVe1L*, *LsVe3L*, and *LsVe4L*).

Only two out of 36 romaine accessions were resistant to the disease in field experiments. One of the resistant accessions, cultivar Annapolis, is a dark red lettuce with a relatively small and light head that is usually grown for baby leaf production and is therefore harvested at early stages of development. The other resistant cultivar was Defender, which is green. Origin of resistance in this cultivar is unknown because it was developed through open pollination [23]. A high frequency (87.5%) of resistance to the disease was found in Latin type accessions that phenotypically resemble a small romaine lettuce with more pliable and oily leaves. Because of the phenotypic similarity between romaine and Latin types, Latin-type accessions may also be used for a relatively rapid development of romaine cultivars with resistance to *V. dahliae* race 1. Both romaine cultivars and three sequenced Latin cultivars (Eruption, Pavane, and Little Gem) that are resistant to the disease contain an identical combination of LsVe alleles (*LsVe1L*, *LsVe3L*, and *LsVe4L*).

Substantially different frequencies of *LsVe1L* alleles (Table 3) and resistant phenotypes (Table 1) in different horticultural types of lettuce are not unexpected considering that comparable differences were previously described for other monogenically inherited traits, such as resistance to lettuce dieback [24] and sensitivity to triforine [25]. Differences in the frequency of specific alleles among horticultural types are likely caused by the breeding approach that is used to develop lettuce cultivars. Only a few elite progenitors or founder lettuce cultivars have given rise to most of the modern commercial cultivars [26]. Each of these progenitors is frequently found in pedigrees of cultivars of the same horticultural type. Additionally, new cultivars are mainly developed by recurrent breeding within small pools of closely related germplasm of the same type [27]. Therefore, alleles present in an original progenitor(s) of a certain type are found in high frequency in cultivars of the same type, but may be absent or present in low frequency in cultivars of other horticultural types.

Our data are consistent with the *LsVe1* gene identified in the cultivar La Brillante being involved in resistance to *V. dahliae* race 1 in lettuce. Among the 152 accessions included in this study, 21 were resistant to *V. dahliae* race 1 and all 21 contained the *LsVe1L* allele; this allele was not present in any of the susceptible accessions. The other La Brillante *Ve* alleles, *LsVe3L* and *LsVe4L*, were also present in all the resistant accessions, but they also occurred in two and twelve susceptible accessions, respectively. Therefore, *LsVe1L* is the strongest candidate as being required for resistance to *V. dahliae* race 1 in lettuce, although our data do not exclude *LsVe3L* or *LsVe4L* from also being involved similarly as in tomato [12]. Complementation and knock-out studies are still required to determine the functional basis of *LsVe-*mediated resistance to *V. dahliae* race 1.

The function and the significance of the differences between the *LsVe1L* and *LsVe1S* alleles (Additional file 7) remains to be investigated. The proteins encoded by *LsVe1L* and *LsVe1S* have the same domain organization, including the 37 extracellular, leucine-rich repeats separated by a short spacer region, as in previously characterized functional Ve proteins in other species [11, 18]. However, in addition to sequence diversity in the extracellular LRR domain, LsVe1L has an additional C-terminal transmembrane domain as compared to Ve1 and Ve2 in tomato, suggesting that maybe LsVe1L crosses the membrane three times and terminates with a non-cytoplasmic domain instead of a cytoplasmic domain.

The distribution of disease incidence in susceptible accessions (from 0.07 to 1.00) and across horticultural types (> 0.98 in stem types, but only 0.17 in a single susceptible Latin) indicates a possible presence of a modifying factor or factors that affect disease incidence. Our data do not exclude the possibility of interactions between two or more *Ve* genes, similar to those reported in tomato [12]. A more detailed study of accessions with different frequencies of disease incidence and allelic compositions is needed to elucidate the basis of variation in disease incidence.

## Conclusions

There is a critical lack of iceberg and romaine type cultivars with resistance to *V. dahliae* race 1. Application of molecular markers can accelerate the lettuce breeding process while improving selection accuracy [28]. Therefore, the development of molecular marker assays for identification of desired genotypes is highly sought-after. The *LsVe1L-*specific PCR assay developed in this study can be used for the selection of lettuce genotypes with resistance to *V. dahliae* race 1. Application of this assay allows identification of resistant genotypes in early stages of plant development (or at a seed-level) without time- and labor-intensive testing of plants in the field. This molecular marker is a valuable addition to the tools available to breeders when developing improved cultivars of lettuce.

## Methods

### Lettuce accessions used for genome sequencing and PCR analysis

A total of 152 lettuce accessions representing all major types of cultivated lettuce (Batavia, butterhead, iceberg, Latin, leaf, oil, romaine, and stem) were analyzed (Table 2). The majority of accessions (111) were from the United States Department of Agriculture, Agricultural Research Service (USDA-ARS) lettuce collections at Salinas, California; the remaining accessions were from a variety of sources (Table 2), including Salinas, the previously sequenced cultivar [16]. When an accession was obtained from more than one source, each provenance was considered separately in the analyses.

### Pathogenicity tests

Experiments were conducted in a field infested with *V. dahliae* race 1 [16] located at the USDA-ARS station in Salinas, California. One hundred and fifty accessions were direct-seeded in a randomized complete block design with three replications. The original seed batches of previously sequenced cultivars Salinas [17] and La Brillante (this publication) were not available for field tests; therefore, seed batches used in field tests are shown as separate entries (Table 2). Each plot was 7 m long and consisted of two seed lines on 1 m wide beds standard for lettuce production in coastal California. Plant spacing was approximately 28 cm between seed lines and 30 cm between plants within a seed line. All field experiments were maintained using standard cultural practices for coastal California lettuce production. Plants were evaluated for disease incidence after reaching harvest maturity. Unless indicated otherwise, ten plants from each plot were uprooted and visually evaluated. Disease incidence was assessed by cutting taproots longitudinally and recording the number of plants exhibiting the yellowish-brown discoloration of root vascular tissues that is typical of Verticillium wilt. Absence of *V. dahliae* race 1 in cultivars with race 1-resistant genotype was confirmed by plating surface-sterilized symptomatic root tissue on NP-10 semi-selective agar medium [29] and PCR screening any resulting isolates with *Ave1*-specific primers [8]. Three additional experiments were performed in the same field to confirm phenotypic observations. These experiments comprised only a subset of accessions that were either symptomless in the first experiment or were used as susceptible checks. Disease incidence values from all four experiments were combined and used for statistical analyses with JMP 14.2 (SAS Institute, Cary, NC, USA).

### DNA extraction

DNA was extracted using FastDNA SPIN kit (MP Biomedicals, Solon, OH, USA) for most lettuce accessions and with the CTAB method [30] for La Brillante accession 10G364–1. For the FastDNA SPIN kit method, up to 100 mg seeds (~ 100 seeds) or freeze-dried leaf tissue was crushed in liquid nitrogen with a mortar and pestle, and DNA was extracted according to the manufacturer’s instructions for plant material. DNA quality was assessed using gel electrophoresis (0.7% agarose gel), a NanoDrop spectrophotometer, and a Qubit Fluorometer (both Thermo Fisher Scientific, Wilmington, DE, USA) as per the manufacturers’ instructions. DNA extraction for *V. dahliae* followed the same FastDNA SPIN kit protocol except that CLS-Y solution was used as suggested by the manufacturer.

### Genome sequencing and assembly

For La Brillante accession 10G364–1, three genomic libraries were constructed, one with 180 bp insert size (with in-house protocols) and two Nextera (Illumina, San Diego, CA, USA) 2 and 7 kb mate-pair libraries. All libraries were sequenced in an Illumina Hi-Seq 2000 for 100 + 100 paired-end reads. Reads were directly imported into AllPaths-LG v49856 [31] and assembled using default parameters. Both mate-pair libraries were aligned to the AllPaths-LG assembly using BWA v0.7.4 [32] and these alignments were fed into SSpace v3.0 [33] together with the assembly to perform further scaffolding.

For the remaining accessions, DNA was sent to Novogene (Beijing, China) for library preparation (insert size 350 bp) and sequencing on Illumina HiSeq 4000 machines to generate ~ 800 M PE150 reads that provided approximately 25x whole genome coverage. Reads obtained from Novogene were further processed to remove low quality sequences using bbduk from the BBMap suite v33.65 [34]. This removed sequences with a quality score below 20 from both ends of the read and eliminated reads that had less than 50 bp after trimming. Genome assemblies were generated using MEGAHIT version 1.0.6 [35] or MaSuRCA version 2.3.2 [36]. MEGHIT was generally run with default settings and sometimes with meta-sensitive or bulk options in effect. MaSuRCA settings were insert size = 350 and standard deviation = 50, GRAPH_KMER_SIZE = 101, USE_LINKING_MATES = 0, LIMIT_JUMP_COVERAGE = 300, CA_PARAMETERS = cgwErrorRate = 0.15 ovlMemory = 4 GB, KMER_COUNT_THRESHOLD = 1, NUM_THREADS = 40, JF_SIZE = 10,000,000,000, and DO_HOMOPOLYMER_TRIM = 0. Assembly statistics were generated using the shell script stats.sh of BBMap version 37.68 [34].

### Ve gene identification and naming

The expressed sequence tag marker QGD8I16.yg.ab1 at the *Verticillium resistance 1* (*Vr1*) locus in lettuce [16] that has sequence similarity to the *Ve* genes of tomato was used to query the genome assemblies of *V. dahliae* race 1 susceptible cultivar Salinas [17] (assembly version 8, available at https://genomevolution.org/coge/GenomeInfo.pl?gid=28333) and race 1 resistant cultivar La Brillante, using local nucleotide BLAST v. 2.6 [37]. The *LsVe* sequences from cultivars La Brillante and Salinas were then used to query the remaining lettuce genome assemblies using BLASTn. Sequence alignments were generated with MAFFT version 7.309 [38, 39] using default settings. Phylogenetic analyses were performed with PAUP 4.0a (build 159) [40] using the maximum parsimony criterion, the heuristic search option, and 10 random addition replicates. Bootstrap branch support was based on 1000 random addition replicates. Default settings were used otherwise. Protein domains were annotated using the InterPro website (https://www.ebi.ac.uk/interpro/) [41]. Codon alignments were subjected to calculation of synonymous and non-synonymous substitution rates with PAL2NAL v. 14 [42].

### PCR assays

La Brillante *LsVe1L* and *LsVe4L*-specific PCR assays were performed as follows. Each assay was a multiplex assay with two *LsVeL*-specific primers and a plant DNA control with two additional primers specific to the lettuce 4-hydroxyphenylpyruvate dioxygenase-encoding gene (*HPPD*), which has been used as the reference gene in real-time PCR assays [43]. PCR conditions and primer sequences are shown in Table 4.

**Table 4:** PCR conditions and primer sequences used for *LsVe1L* and *LsVe4L* multiplex assays

Target locus^a^	Forward primer^b^	Reverse primer^b^	Annealing temperature^c^	Amplicon size^d^
*LsVe1L*	5′-CAA GGG CTC TAT GTC ATT CCT CC	5′-GAC CCA TGG AAG CTG TTG GAT CT	60 °C	569 bp
*LsVe4L*	5′-CTT GTC CCA GAT AGA GTT GTC CAC C	5′-CAG ACC CTG GAA ATC TTT GGT TTG A	57 °C	505 bp
*HPPD* ^1^	5′-TCC CAA CTC CTC ACA CTC CTT AAT C	5′-GTA CGG AAC AAA GAG GAA GAG CC	57 °C or 60 °C	244 bp

^a^The lettuce *HPPD* was targeted as a DNA quality control in both the *LsVe1L* and *LsVe4L* multiplex assays

^b^Each 25 μL PCR reaction contained 1.25 μL of each of the four primers at 10 μM each to amplify *HPPD* plus *LsVe1L* or *HPPD* plus *LsVe4L*, 12.5 μL 2x GoTaq Colorless Master Mix (Promega Corp., Madison, WI), and 7.5 μL DNA template from a 1 ng/μL stock

^c^The PCR program consisted of an initial denaturation at 94 °C for 2 min, followed by 32 cycles of denaturation at 94 °C for 10 s, 20 s at the assay-specific annealing temperature, extension at 72 °C for 1 min, and a final extension at 72 °C for 7 min. PCRs were set up on ice under sterile conditions and the thermocycler was preheated to 94 °C before adding the reactions

^d^PCR products (8 μL each) were run on a 1% agarose gel

## Additional files

Additional file 1: Alignment of tomato *Ve1* and lettuce *LsVe* alleles. Domains are indicated; eLRR stands for extracellular leucine-rich repeat. Domain information for *Ve1* is from [18]. (PDF 589 kb)

Additional file 2: *LsVe* alleles found in this study. Provided are names of contigs or scaffolds, identical representatives included in Fig. 4, and completeness of sequencing coverage. (XLSX 15 kb)

Additional file 3: Phylogenetic tree of cultivars La Brillante and Salinas *Ve* allele amino acid sequences and homologs from other plant families using maximum parsimony. One of two most parsimonious trees is shown measuring 3492 steps; the tree is midpoint rooted. Taxa names consist of species names followed by gene names. GenBank accession numbers are provided for sequences from other studies. Bootstrap supports above 60% are shown by the branches. Branch lengths are proportional to changes along the branches and the scale is provided. (PDF 16 kb)

Additional file 4: Results of *LsVe1L* and *LsVe4L* PCR screening of 90 lettuce accessions that were not genome sequenced. The legend to lane numbers is in Additional file 5. For each accession, the top gel shows results of the *LsVe1L* screening, the bottom gel shows the results of the *LsVe4L* screening. Resistant accessions are marked with an R. Amplicon sizes are indicated by > and correspond to 200 and 500 bp. Size standard used is 2-log ladder. PCR conditions are described in Table 4. (PDF 1577 kb)

Additional file 5: Legend to lane numbers in Additional file 4. For each lane, accession name, code, and PCR result are provided. (XLSX 12 kb)

Additional file 6: PCR gel demonstrating that *Verticillium dahliae* strains isolated from symptomatic cultivar Plymouth tap roots did not contain *Ave1*, the specificity determinant of race 1, and were thus not race 1. Amplicon size marker indicated by > corresponds to 1000 bp. Lane numbers are: 1. 2-log ladder, 2. and 3. *Verticillium dahliae* strain isolated from symptomatic cultivar Plymouth tap root, 4. and 5. *V. dahliae* race 2 control strain Ls.17, 6. and 7. *V. dahliae* race 1 control strain Ls.16, and 8. negative control. (PDF 4236 kb)

Additional file 7: Nucleotide sequences of six *LsVe* alleles from cultivars La Brillante (L) and Salinas (S). (DOCX 17 kb)

## Data Availability

Data and results generated and analyzed during this study are included in this published article and its supplementary information files. Nucleotide sequences of six *LsVe* alleles from cultivars La Brillante and Salinas are provided in Additional file 7. Sequence data of 61 genomes generated and analyzed during the current study are available in GenBank at https://www.ncbi.nlm.nih.gov/bioproject/PRJNA478460.

## Abbreviations

*HPPD*: 4-hydroxyphenylpyruvate dioxygenase-encoding gene
*LsVe1*, *LsVe2*, *LsVe3*, and *LsVe4*: *Lactuca sativa* homologs of tomato *Ve* genes for resistance to *Verticillium dahliae* race 1
ORF: Open reading frame
RLP: Receptor-like proteins
SNP: Single nucleotide polymorphisms
*Vr1*: *Verticillium resistance 1* locus

## References

[1] Simko I, Hayes RJ, Mou B, McCreight JD, Smith S, Diers B, Specht J, Carver B (2014). Lettuce and spinach. Yield gains in major US field crops.

[2] Atallah ZK, Hayes RJ, Subbarao KV (2011). Fifteen years of Verticillium wilt of lettuce in America's salad bowl: a tale of immigration, subjugation, and abatement. Plant Dis.

[3] Inderbitzin P, Subbarao KV, Subbarao KV, Davis RM, Gilbertson RL, Raid RN (2017). Verticillium wilt. Compendium of lettuce diseases and pests.

[4] Pegg GF, Brady BL (2002). Verticillium wilts.

[5] Inderbitzin P, Subbarao KV (2014). *Verticillium* systematics and evolution: how confusion impedes Verticillium wilt management and how to resolve it. Phytopathology.

[6] Vallad GE, Qin Q-M, Grube R, Hayes RJ, Subbarao KV (2006). Characterization of race-specific interactions among isolates of *Verticillium dahliae* pathogenic on lettuce. Phytopathology.

[7] Gurung S, Short DPG, Atallah ZK, Subbarao KV (2014). Clonal expansion of *Verticillium dahliae* in lettuce. Phytopathology.

[8] de Jonge R, van Esse PH, Maruthachalam K, Bolton MD, Santhanam P, Saber MK (2012). Tomato immune receptor Ve1 recognizes effector of multiple fungal pathogens uncovered by genome and RNA sequencing. Proc Natl Acad Sci U S A.

[9] Kawchuk LM, Hachey J, Lynch DR, Kulcsar F, van Rooijen G, Waterer DR (2001). Tomato *Ve* disease resistance genes encode cell surface-like receptors. Proc Natl Acad Sci U S A.

[10] Wang G, Fiers M, Ellendorff U, Wang Z, de Wit PJ, Angenent GC (2010). The diverse roles of extracellular leucine-rich repeat-containing receptor-like proteins in plants. Crit Rev Plant Sci.

[11] Song Y, Zhang Z, Seidl MF, Majer A, Jakse J, Javornik B (2017). Broad taxonomic characterization of Verticillium wilt resistance genes reveals an ancient origin of the tomato Ve1 immune receptor. Mol Plant Pathol.

[12] Nazar Ross N., Xu Xin, Kurosky Alexander, Robb Jane (2018). Antagonistic function of the Ve R-genes in tomato. Plant Molecular Biology.

[13] Chen J, Li N, Ma X, Gupta VK, Zhang D, Li T, et al. The ectopic overexpression of the cotton *Ve1* and *Ve2*-homolog sequences leads to resistance response to Verticillium wilt in Arabidopsis. Front Plant Sci. 2017;8:844. 10.3389/fpls.2017.00844 PMC5447073 28611793

[14] Simko I, Costanzo S, Haynes KG, Christ BJ, Jones RW (2004). Linkage disequilibrium mapping of a *Verticillium dahliae* resistance quantitative trait locus in tetraploid potato (*Solanum tuberosum*) through a candidate gene approach. Theor Appl Genet.

[15] Simko I, Haynes KG, Ewing EE, Costanzo S, Christ BJ, Jones RW (2004). Mapping genes for resistance to *Verticillium albo-atrum* in tetraploid and diploid potato populations using haplotype association tests and genetic linkage analysis. Mol Gen Genomics.

[16] Hayes R, McHale L, Vallad G, Truco M, Michelmore R, Klosterman S (2011). The inheritance of resistance to Verticillium wilt caused by race 1 isolates of *Verticillium dahliae* in the lettuce cultivar La Brillante. TAG Theor Appl Genet.

[17] Reyes-Chin-Wo S, Wang Z, Yang X, Kozik A, Arikit S, Song C, et al. Genome assembly with *in vitro* proximity ligation data and whole-genome triplication in lettuce. Nat Commun. 2017;8:14953. 10.1038/ncomms14953 PMC5394340 28401891

[18] Zhang Z, Song Y, Liu C-M, Thomma BPHJ (2014). Mutational analysis of the Ve1 immune receptor that mediates *Verticillium* resistance in tomato. PLoS One.

[19] Hayes RJ, Vallad GE, Qin Q-M, Grube RC, Subbarao KV (2007). Variation for resistance to Verticillium wilt in lettuce *(Lactuca sativa* L.). Plant Dis.

[20] Hayes Ryan J., Sandoya German, Mou Beiquan, Simko Ivan, Subbarao Krishna V. (2018). Release of Three Iceberg Lettuce Populations with Combined Resistance to Two Soilborne Diseases. HortScience.

[21] Hayes Ryan J., Maruthachalam Karunakaran, Vallad Gary E., Klosterman Steven J., Simko Ivan, Luo Yaguang, Subbarao Krishna V. (2011). Iceberg Lettuce Breeding Lines with Resistance to Verticillium Wilt Caused by Race 1 Isolates of Verticillium dahliae. HortScience.

[22] Simko Ivan, Hayes Ryan J., Bull Carolee T., Mou Beiquan, Luo Yaguang, Trent Mark A., Atallah Amy J., Ryder Edward J., Sideman Rebecca G. (2014). Characterization and Performance of 16 New Inbred Lines of Lettuce. HortScience.

[23] Wehner Todd C. (2002). Vegetable Cultivar Descriptions for North America List 26 2002. HortScience.

[24] Simko I, Pechenick DA, McHale LK, Truco MJ, Ochoa OE, Michelmore RW (2009). Association mapping and marker-assisted selection of the lettuce dieback resistance gene *Tvr1*. BMC Plant Biol.

[25] Simko I, Hayes RJ, Truco MJ, Michelmore RW (2011). Mapping a dominant negative mutation for triforine sensitivity in lettuce and its use as a selectable marker for detecting hybrids. Euphytica.

[26] Mikel Mark A. (2007). Genealogy of Contemporary North American Lettuce. HortScience.

[27] Mikel MA (2013). Genetic composition of contemporary proprietary US lettuce (*Lactuca sativa* L.) cultivars. Genet Resour Crop Evol.

[28] Simko I. (2013). Marker-Assisted Selection for Disease Resistance in Lettuce. Translational Genomics for Crop Breeding.

[29] Kabir Z, Bhat RG, Subbarao KV (2004). Comparison of media for recovery of *Verticillium dahliae* from soil. Plant Dis.

[30] Doyle J, Doyle JL (1987). Genomic plant DNA preparation from fresh tissue-CTAB method. Phytochem Bull.

[31] Butler J., MacCallum I., Kleber M., Shlyakhter I. A., Belmonte M. K., Lander E. S., Nusbaum C., Jaffe D. B. (2008). ALLPATHS: De novo assembly of whole-genome shotgun microreads. Genome Research.

[32] Li H, Durbin R (2010). Fast and accurate long-read alignment with burrows–wheeler transform. Bioinformatics.

[33] Boetzer M, Henkel CV, Jansen HJ, Butler D, Pirovano W (2010). Scaffolding pre-assembled contigs using SSPACE. Bioinformatics.

[34] Bushnell B: BBMap. Retrieved from sourceforge.net/projects/bbmap**/**. 2016.

[35] Li D, Liu C-M, Luo R, Sadakane K, Lam T-W (2015). MEGAHIT: an ultra-fast single-node solution for large and complex metagenomics assembly via succinct de Bruijn graph. Bioinformatics.

[36] Zimin AV, Marçais G, Puiu D, Roberts M, Salzberg SL, Yorke JA (2013). The MaSuRCA genome assembler. Bioinformatics.

[37] Altschul SF, Gish W, Miller W, Myers EW, Lipman DJ (1990). Basic local alignment search tool. J Mol Biol.

[38] Katoh K, Misawa K, Ki K, Miyata T (2002). MAFFT: a novel method for rapid multiple sequence alignment based on fast Fourier transform. Nucleic Acids Res.

[39] Katoh K, Standley DM (2013). MAFFT multiple sequence alignment software version 7: improvements in performance and usability. Mol Biol Evol.

[40] Swofford DL (2002). PAUP*. Phylogenetic analysis using parsimony (*and other methods). Version 4.

[41] Finn RD, Attwood TK, Babbitt PC, Bateman A, Bork P, Bridge AJ (2017). InterPro in 2017—beyond protein family and domain annotations. Nucleic Acids Res.

[42] Suyama M, Torrents D, Bork P (2006). PAL2NAL: robust conversion of protein sequence alignments into the corresponding codon alignments. Nucleic Acids Res.

[43] Borowski JM, Galli V, da Silva Messias R, Perin EC, Buss JH, e Silva SDA (2014). Selection of candidate reference genes for real-time PCR studies in lettuce under abiotic stresses. Planta.

