# Correction to: The *LsVe1L* allele provides a molecular marker for resistance to *Verticillium dahliae* race 1 in lettuce

**DOI:** 10.1186/s12870-019-1966-9

**Published:** 2019-08-26

**Authors:** Patrik Inderbitzin, Marilena Christopoulou, Dean Lavelle, Sebastian Reyes-Chin-Wo, Richard W. Michelmore, Krishna V. Subbarao, Ivan Simko

**Affiliations:** 1 Department of Plant Pathology, University of California, Davis, CA 95616 USA; 2 Present address: Indigo Ag, Charlestown, MA 02129 USA; 3 Genome Center, University of California, Davis, CA 95616 USA; 4 Departments of Plant Sciences, Molecular & Cellular Biology, Medical Microbiology & Immunology, University of California, Davis, CA 95616 USA; 5 United States Department of Agriculture, Agricultural Research Service, Crop Improvement and Protection Research Unit, Salinas, CA 93905 USA

**Correction to: BMC Plant Biol (2019) 19:305**

**https://doi.org/10.1186/s12870-019-1905-9**

Following publication of the original article [1], the author reported a processing error in Fig. 5. This has been corrected in the original article.

The correct version of Fig. 5 is also shown in this correction.

**Fig. 5:**
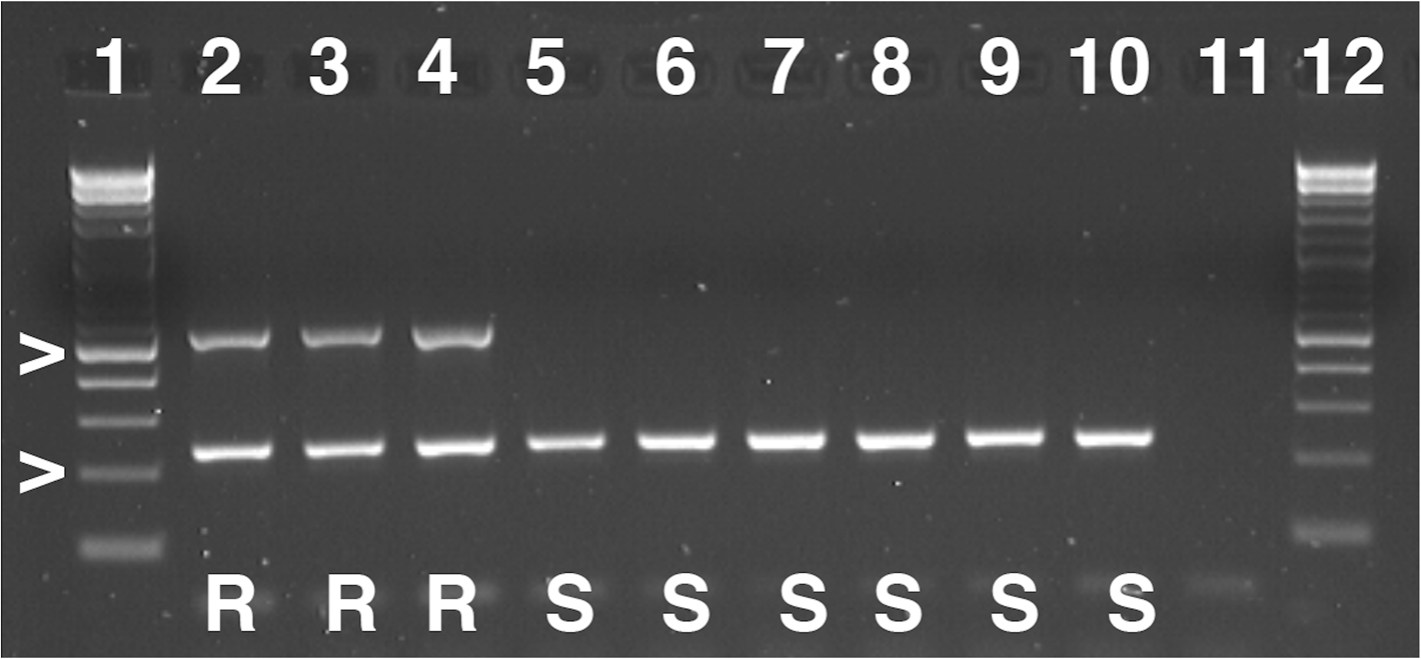
*LsVe1L* specific PCR assay is allele-specific. Shown are results of *LsVe1L-*specific PCR assays with selected lettuce accessions with known *LsVe* genotypes and resistance phenotypes. Resistance (R) and susceptibility (S) is indicated by capital letters for each accession. In all cases, the outcomes of the PCR assays were as expected from genome sequencing. Amplicon sizes are indicated by > and correspond to 200 and 500 bp. Lane numbers are: 1. 2-log ladder, 2. cultivar Balady Banha (*Ve* genotype: *LsVe1L, LsVe3L, LsVe4L*), 3. cultivar Lolla Rossa (*LsVe1L, LsVe3L, LsVe4L*), 4. cultivar Plymouth (*LsVe1L, LsVe3L, LsVe4L*), 5. cultivar Cobham Green (*LsVe3L, LsVe4L, LsVe1S, LsVe2S*), 6. cultivar Lee Tal (*LsVe4L, LsVe1S, LsVe2S*), 7. cultivar Margarita (*LsVe4L, LsVe1S, LsVe2S*), 8. cultivar Anuenue (*LsVe2S, LsVe3S*), 9. cultivar Blonde Lente a Monter (*LsVe1S, LsVe2S, LsVe3S*), 10. cultivar Primus (*LsVe1S, LsVe2S, LsVe3S*), 11. negative control, and 12. 2-log ladder. PCR conditions are described in Table 4

In the Additional file 7, the sequence data for LsVe3L and LsVe4L were swapped. The correct Additional file 7 is shown in the Additional file section below.

## Additional file

Additional file 7: Nucleotide sequences of six LsVe alleles from cultivars La Brillante (L) and Salinas (S). (DOCX 17.8 kb)
