# Supplementary figures and images for: The LsVe1L allele provides a molecular marker for resistance to Verticillium dahliae race 1 in lettuce
Source: BMC Plant Biol. 2019 Jul 10;19:305. doi: 10.1186/s12870-019-1905-9 (PMC6621938; doi:10.1186/s12870-019-1905-9)

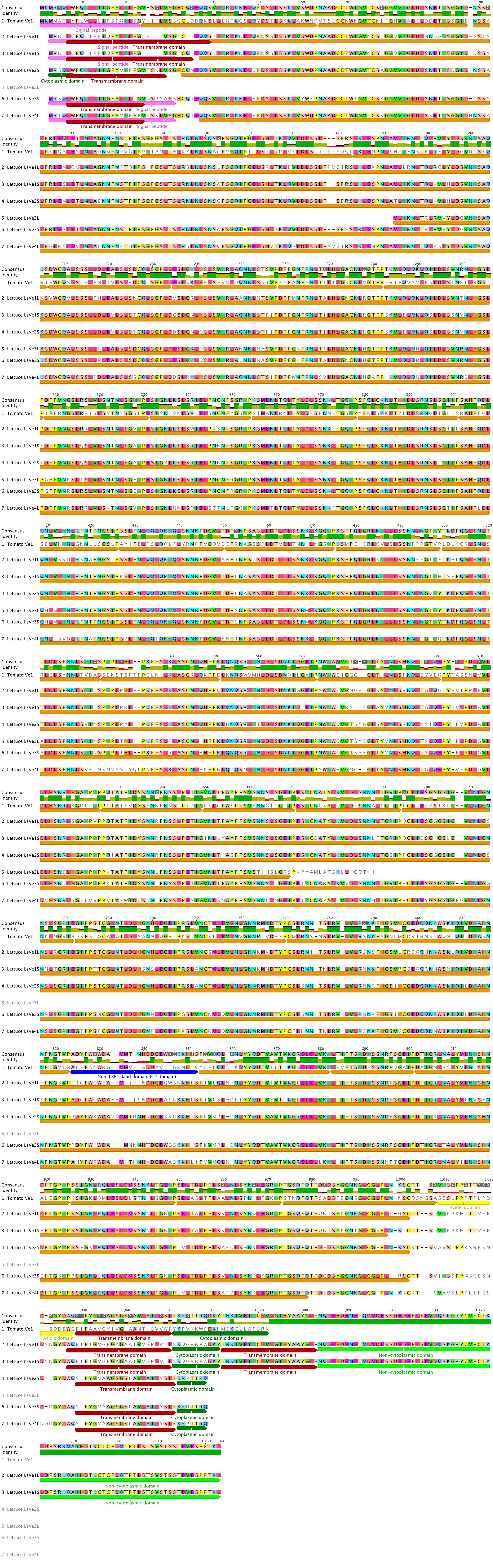

Supplement: Supplementary file 1 — Alignment of tomato Ve1 and lettuce LsVe alleles. Domains are indicated; eLRR stands for extracellular leucine-rich repeat. Domain information for Ve1 is from [18]. (PDF 589 kb) [file 12870_2019_1905_MOESM1_ESM.pdf]

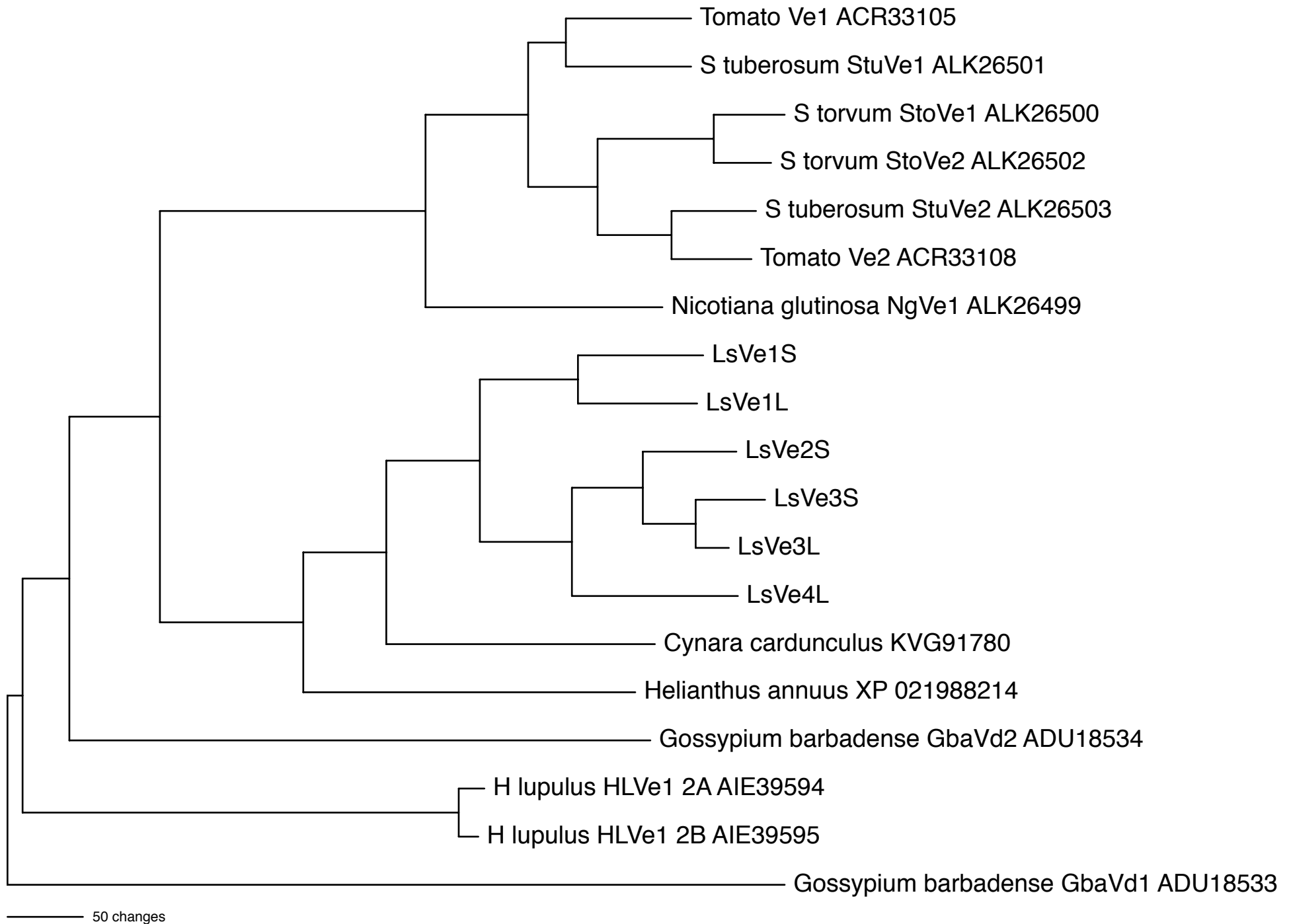

Supplement: Supplementary file 3 — Phylogenetic tree of cultivars La Brillante and Salinas Ve allele amino acid sequences and homologs from other plant families using maximum parsimony. One of two most parsimonious trees is shown measuring 3492 steps; the tree is midpoint rooted. Taxa names consist of species names followed by gene names. GenBank accession numbers are provided for sequences from other studies. Bootstrap supports above 60% are shown by the branches. Branch lengths are proportional to changes along the branches and the scale is provided. (PDF 16 kb) [file 12870_2019_1905_MOESM3_ESM.pdf]

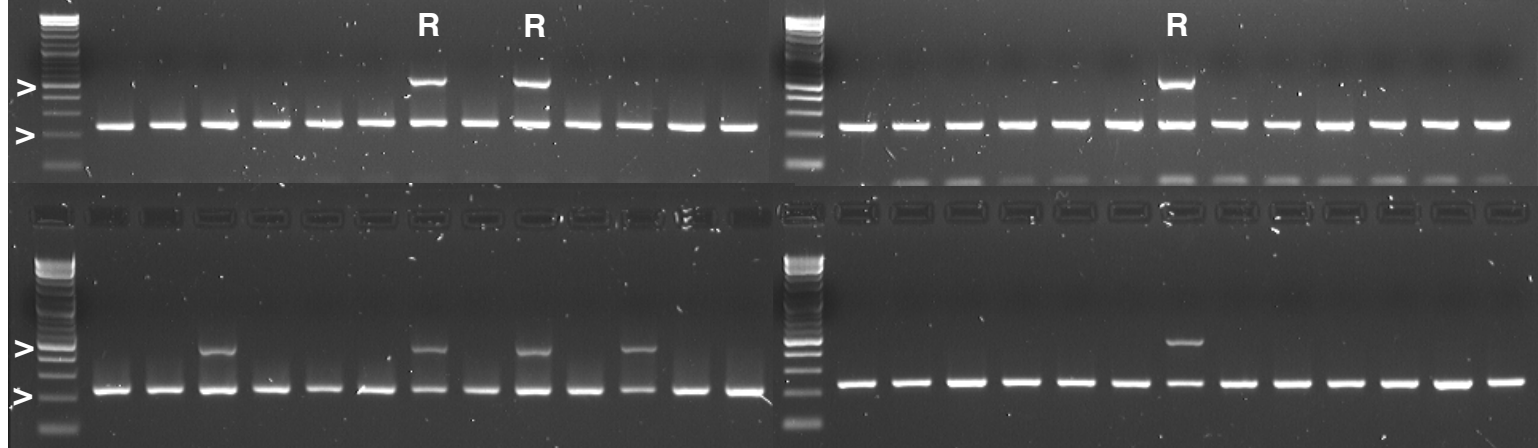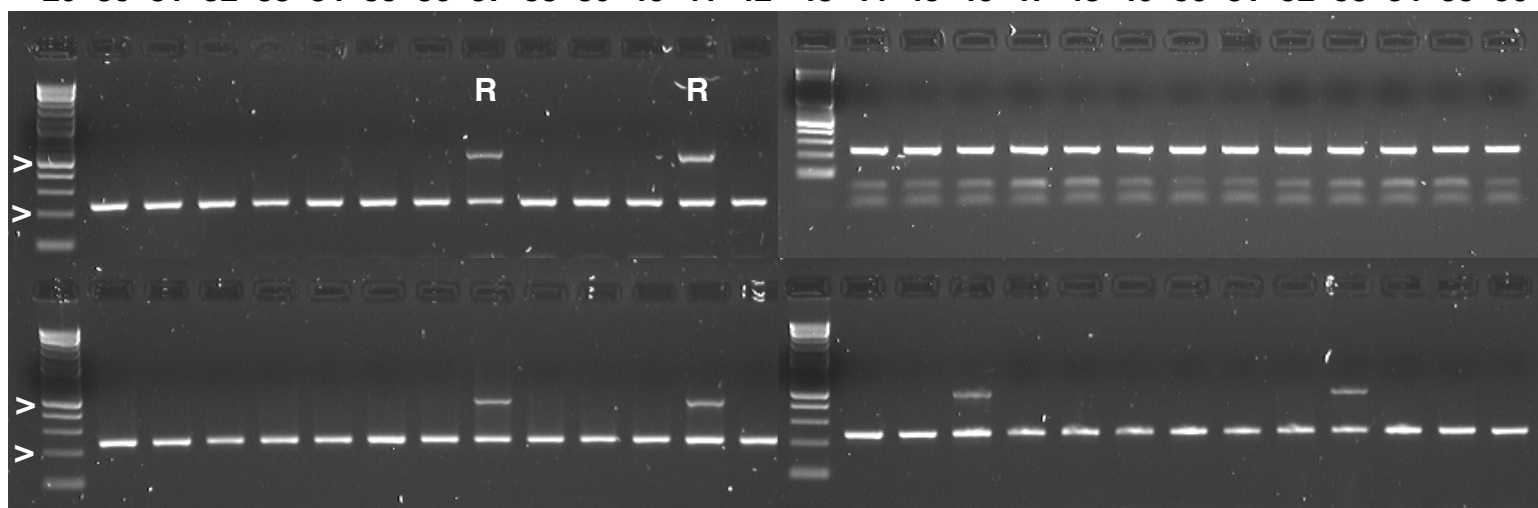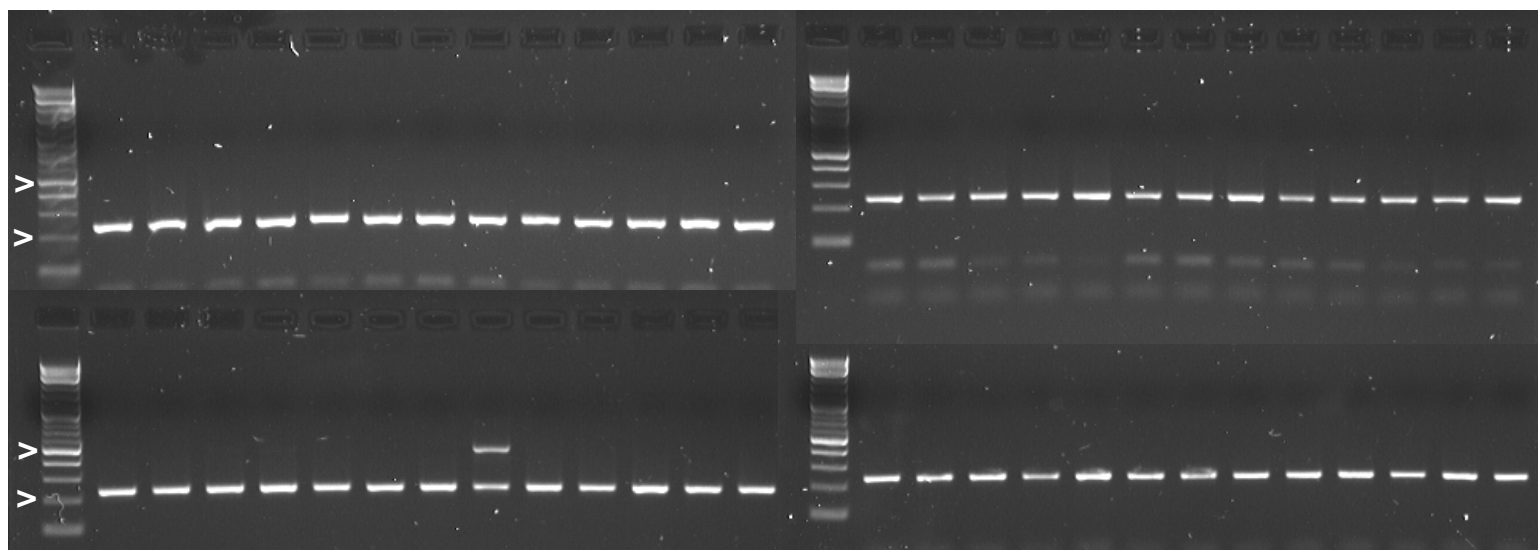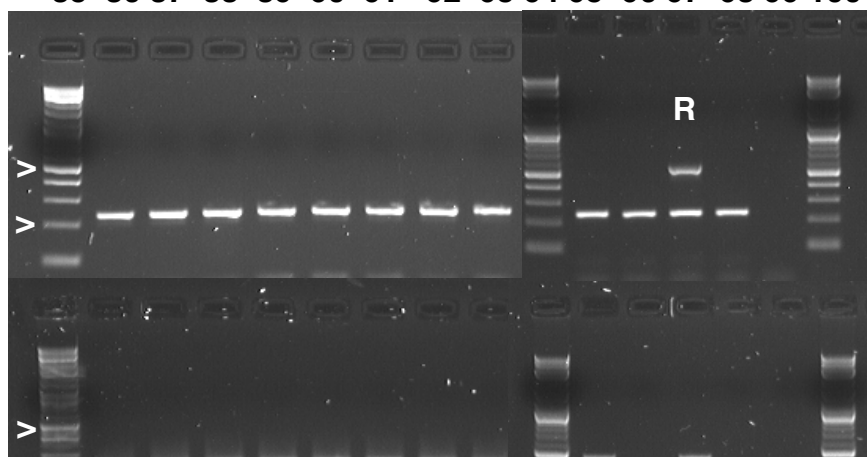

Supplement: Supplementary file 4 — Results of LsVe1L and LsVe4L PCR screening of 90 lettuce accessions that were not genome sequenced. The legend to lane numbers is in Additional file 5. For each accession, the top gel shows results of the LsVe1L screening, the bottom gel shows the results of the LsVe4L screening. Resistant accessions are marked with an R. Amplicon sizes are indicated by > and correspond to 200 and 500 bp. Size standard used is 2-log ladder. PCR conditions are described in Table 4. (PDF 1577 kb) [file 12870_2019_1905_MOESM4_ESM.pdf]

1

2

3

4

5

6

7

8

V

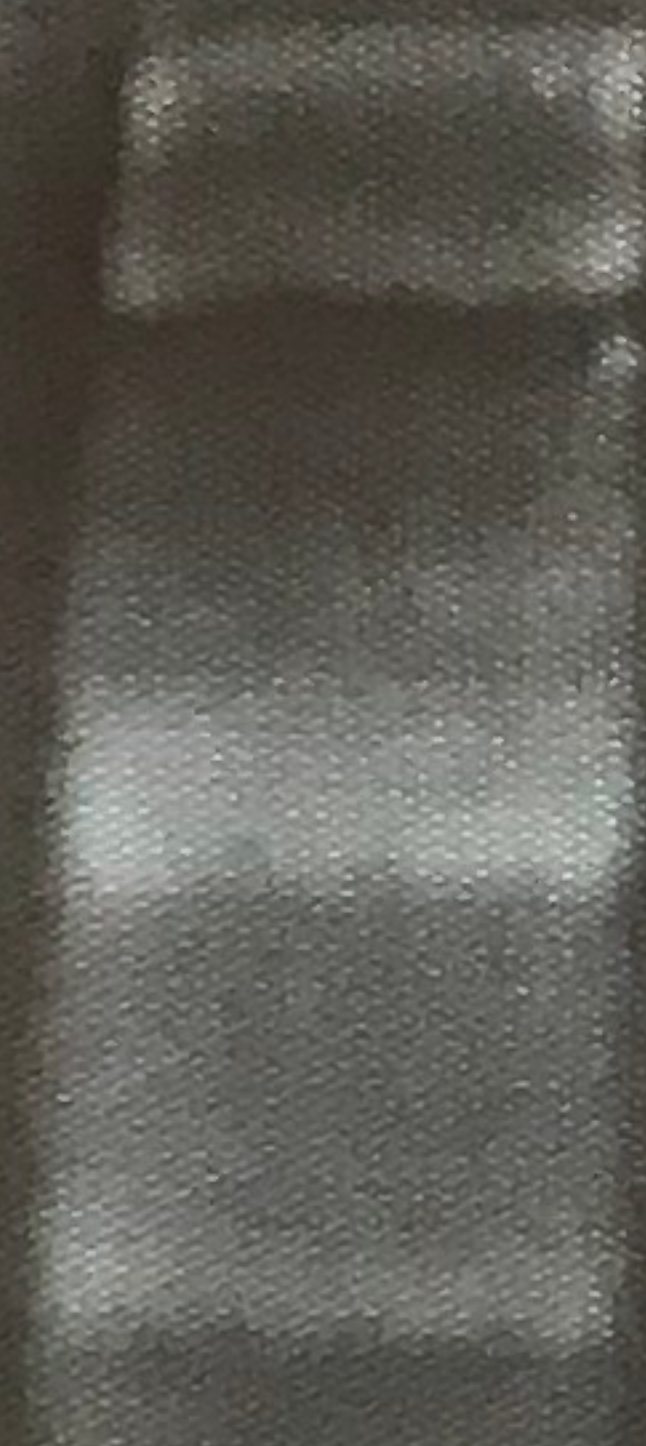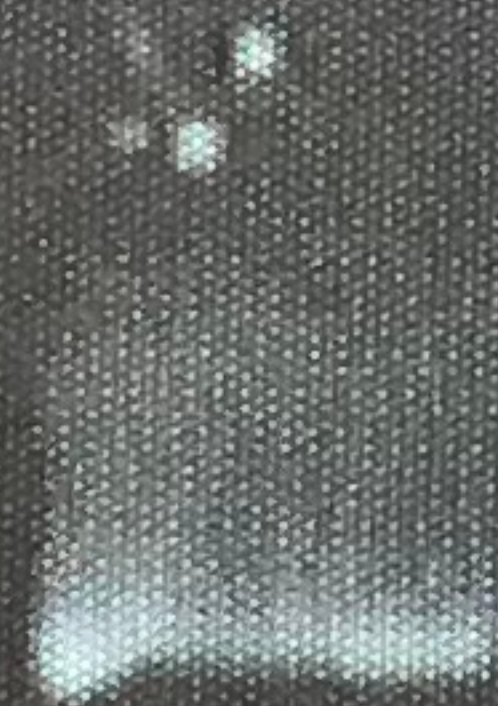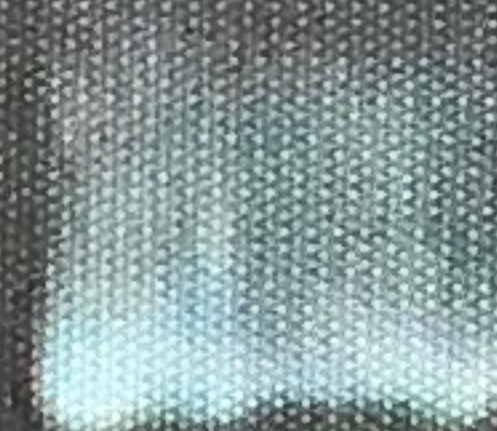

Supplement: Supplementary file 6 — PCR gel demonstrating that Verticillium dahliae strains isolated from symptomatic cultivar Plymouth tap roots did not contain Ave1, the specificity determinant of race 1, and were thus not race 1. Amplicon size marker indicated by > corresponds to 1000 bp. Lane numbers are: 1. 2-log ladder, 2. and 3. Verticillium dahliae strain isolated from symptomatic cultivar Plymouth tap root, 4. and 5. V. dahliae race 2 control strain Ls.17, 6. and 7. V. dahliae race 1 control strain Ls.16, and 8. negative control. (PDF 4236 kb) [file 12870_2019_1905_MOESM6_ESM.pdf]
