# Supplementary material for: The LsVe1L allele provides a molecular marker for resistance to Verticillium dahliae race 1 in lettuce
Source: BMC Plant Biol. 2019 Jul 10;19:305. doi: 10.1186/s12870-019-1905-9 (PMC6621938; doi:10.1186/s12870-019-1905-9)
Supplement: Supplementary file 7 — Nucleotide sequences of six LsVe alleles from cultivars La Brillante (L) and Salinas (S). (DOCX 17 kb) [file 12870_2019_1905_MOESM7_ESM.docx]

**Nucleotide sequences of six *LsVe* alleles from cultivars La Brillante (L) and Salinas (S).**

>LsVe1L

ATGAGAAATCACCTCTGTTTTCAGATTTTCTTCATACAGTTTTACTTAATTTTATTTGGAGTTTCTGGTATATGTGAAATCGAGCAGCAATCGATTTTGATCCGGTTGAAGAACGAATTACAGTTCAATTCTTCGTTATCATCAAAATTAGTGTCTTGGGACCCAAATGCAGCAGATTGCTGCACCTGGATAGGTGTTAATTGCAGCATCGGGGGTCAGGTTATCGGCCTAGATTTAAACAACGACGCTATATCTGGTGGTATTGATGGTTCTACTTCTCTTTTCCGTTTAGAGAATCTTCAGATGCTGAATCTGGCTGGAAATAACTTCAATTTCACACAGATTCCTTCGATATTTGGCAGTCTGACTAGTTTGAGGAGTTTGAACTTGTCAAATTCGTTGTTTTCAGGGCAGATTCCAGGAGAATTGTCACGACTGACAAAGCTTGAAGTTCTTGATTTATCTTCGCTTTTCCCCATGGGAATTCGCTCACTGAAACTTGAGAAACCCAATCTAGCCATGCTTCTTAGGAACCTCACACAACTTAGAGGTCTTTATCTGGATAGTGTGAACATATCAGCACAAAACTCTGTTTGGTGCCAGGTTTTATCCTCATCTTTGCCACACTTAGAAGCTTTGAGCTTGTCAAATTGTCAACTTTCAGGCCCTTTAGACGTCTCCCTTGGAAGGCTACACTCCCTCTCTGTAATTCGTCTGGCTGCAAACAATCTTAATACTTCAGTTCCAGATTTTTTTATGAATTTCAGGAATCTGACGGCTTTGCATCTCGGTAATTGTAATTTGCGTGGAACATTTCCTACCAAGGTCCTCCAGTTGCAGAAATTACAGATTCTGGATTTATCTGTTAATACAAATCTTCATGGTTCCTTACCAGATTTTCCTGTGAACGGATCCCTTCGAAGATTAGTGCTCAGCAACACAAACCTTTCAGGGGTGATACCAGAATCTATTGGAAATCTTAAGAGCTTATCCTGGATAGAGCTTCCCAAGAGCAATTTCAGTGGACGAATACCTAAATCCATGGAAAACCTTACCCAGTTGACCTACCTTGACTTGTCATCAAACAAGTTCACCGGCCAAATCCCATCATTTCAGCTGTGTAAAAATCTTACCCATATTGACCTCTCACGGAACAGTTTATCAGGTACCATCCTTTCTGCTCACTTCCAAGATCTTCAAAATCTTGTGTCTGTTGATCTTAGATCTAATGCTTTCAATGGAAGCCTACCTTCGTCTTTGTTCAATCTGCAACAACTGCAAAAGATCCAGCTTTCCAACAACAATTTTGATGGTGTACTTGCTAATTTTACTAATCCATCTACGTCTTTACTGGACACTCTTGATTTAAGCAGTAACAAGCTGAAAGGACAAATTCCCAAATCTTTCTTTCAACTTGGACGGCTTAGTATCCTCTTGCTATCATCAAACAACTTCAGTGGCATAATCGAAACCAAAGAACTCCAGGGTCTGAGCAATCTAACAACCCTTGATCTTTCCTTCAACAACTTGTCAATTATAACGAGCCCAATCCCCTTGCCTCATCTTCCAAAGTTTTTCTCATTAAAGTTGGCTTCATGCAATCTCCAGCATTTCCCTCAACTACAAAACCAATCTAGATTGATAAATCTAGACCTTTCAGACAACAAAATTGGTGGAGAAATACCAAGCTGGATCTGGGAGGTTGGAAATGGAGGCCTTTCATATCTCAACCTTTCTCGTAACCAGTTAACTGGCCTCCAAGGGCTCTATGTCATTCCTCCTCTTTTTGTCCTCGATCTGCATTCCAATCGCCTCAGTGGGGCGATTCCAGTTCCACCACAAACTGCTACCTTCATTGATTACTCCAACAATCGTTTCAATTCCTCACTCCCTGAAACCATTGGGGTAAACCTCACTTTTGCCTATTTCTTTTCGGTTTCTAACAATTCACTCTCTGGGGAGATTCCCGAATCCATCTGCAATGCTACCTACCTCAAGGTTCTTGATTTGTCAAACAACAATTTAACTGGAAGGATACCACAGTGTTTGATTGAATCTGGTGGTGGCAGTATTGGGGTACTCAACTTGGGAAGCAATAGTCTCCGTGGAAGAATCGAGGGAATATTCCCAAGCACATGTGGCCTAAACACTTTAGACCTTCATGGAAACCATTTGGAAGGGGAGATCCCACGTTCTCTTGTCAATTGCAGCATGCTAGAAGTCTTAAACCTTGGGAACAACAAGATGAAAGACACGTATCCTTGCTCTTTGCGTAACATTACAAGTTTGCGTGTCCTTGTTCTGAGATCCAACAGCTTCCATGGGTCTGTGCTTTGTAGGGATCAACAACACAACAAATGGTCAAAGCTTCAGATTGTGGACATAGCTCACAATCACTTCAATGGCTCTGTCCCTACAACTTGTTTCTGGCAATGGGAAGCAATGACAATTAATGATGATGGTGAACAATGGAGCAACAAGCATCTCAGTTTCACAGTGTTACAGCTCAATAACTTGTACTATCAGGATACTGTCACAGTCACTGTCAAAGGACTTGAGTTGGAGCTTGTGAAAATCCTGACAATCTTCACATCCATTGATATCTCCAGCAACCGTTTTTCAGGAGAAATACCAGACACAATTGGGAGACTCAATGCTCTGTATATGCTCAACATATCACATAATGACTTCACAGGCCCTATCCCCTCATCTATCGGGAATTTGAGGCAGCTTGAATCATTAGACATGTCATCAAACAATCTAACTGGAAATATCCCATCAGAGCTCACTGCTCTTCCATTCCTTTCTGTTCTTAATCTATCATACAATCAATTGGAAGGAAGAATCCCAACAGGCTCCCAGTTTCAGACCTTCCAAAACACATCATACAAAGGAAACAAGGGATTATGTGGGTTGCCTCTGAGTAGAAGTTGCACTACTTCAGATGTAATCAAACCAAAGCACACAACAACAGTTTTTGAAGATTCTAATGGATATGATTGGCAGTTTATATTCACAGGAGTGGGATTCGGGGCAGGTGCAGCCATTGTTGTAGGGCCTCTCGTGTTGTCTAAGCAAGGGAGAAACTTCTGGGACAAGTACACAAACAAGATTGTGGAGATTATCTGTCTGGTTTTGGGTATCCATTATGCAGCTTATGGGTTATTCAACCAAGATGAACATGATGAAAATGAGACACAAGACATGGATGAAAGCTCAGATGAAGATGAATTTCTGTCAGAAGTTGATCAGTCTAAAGGTAGGTATTGTGTGTTTTGTACCAAACTTGATTTTTCTAGGAAACAAGCCATTCATGATACAAAATGCACTTGCTTTGACCAAACACCAACATTGTCTACTTCAGTGTCCACTTCTTCAACGGAAGTAGAGTCTCCATTTACCAAATTGTGA

>LsVe3L

ATGAGAACCCAACTATGGTTTCAGATTTTGTTGATAATTGGCTTTTACGTGATCTTTTTTGAGGTCAGTTCCAAACTAGTTTCTGGCCATTGTCAAACCGAGCAGCAATCGGTTTTGATCCGGTTGAAGAAAGAATTACATTTCGATTCTTTGTTATCATCAAAATTAGTGTCATGGGACCCAAATGCAGCAGATTGCTGCACCTGGATAGGTGTTACTTGCAGTGTTGGGGGTGTTGTCATAGGCCTAGATTTAAGCAGCGAGACGATATCTGGTGGCATTGATCATAATTCTAGTGCTCTCTTCGGTTTAAAGTATCTCGAGATGCTGAATCTGGCTGAAAATAACTTCAACTTCACACACATTCCTTCCGGATTTGGAAGTCTGACAAGTTTGAGGAGTTTGAACTTGTCAAATTCGCAGTTTTCAGGCCAGATTCCTGGCGAATTGTCGCATATGACAAAGCTTCAATTTCTTGATCTATCTTCGCTTTTCTCCATGGGAATTCGCTCACTGAAACTTGAGAACCCCAATCTGGCCATGCTTATCAAAAACCTCACACAGCTTAAAAGTCTTTATCTGGATAGCGTGAACATATCAGCCCAAAAATCTGATTGGTGCCAGGCTTTATCCTCTTCTTTGCCTGATTTGGAGGCTTTGAGCTTGTCAAGTTGTCAACTTTCAGGCCCTTTAGATGACTCCCTTCGGAAGCTACACTCACTTTCTGTAATTCGTCTGGCTCAGAACAATCTTAGCACTTCAATTCCAGATTTCTTTTGGAATTTCAGAAATCTGAGTGTTCTTCATCTTGGTGCTTGTAATTTGCATGGAGCATTTCCCAAGGTCCTCCAATTGCAGAATTTACAGATTCTGGATTTATCTGTTAATAGGGATCTTCATGGTTCCTTACCAGATTTTCCTGTCAACAGATCCCTTCGAAGATTAGTGCTCAGCAAAACAAACCTTTCAGGTGTGATACCGGAGTCTATTGGAAATCTTAACAACTTGTCCCAGATAGAGTTGTCCACCACAAATCTTTTTGGGCAAATACCAAAGTCAATGGAAACCCTCACCCAATTGACTTACCTTGACTTGTCATCAAACAAATTCACCGGCCAAATCCCATCCTTTCAGCTTTGCAAGAATCTTACACATATTGATCTCTCACGAAACAGTTTATCGGGTACCATCCCTTCTGCTCACTTCCTCGATCTTCAAAATCTCATCTCTGTTGATCTAAGATTCAATGCTTTCAATGGAAGCATACCTTCACCTCTGTTCAATCTGCAACAAATACAAAAGATACAGCTTTCCAACAACAATTTTGATGGCGTACTTGCTAATTTTACTAATCCATCTGCATCTTTACTGGACACTCTTGATTTGAGCAGTAACAAATTGGAAGGGCAAATTCCCAAGTCTTTCTTTCAACTTGGACGGCTTAATATCCTCTTGCTATCATCAAACAACCTGACTGGCTTTATTCAAACCAAAGATTTCCAGGGTCTGAGCAACCTAACGACACTTGATCTTTCATTCAACAACTTGTCAGTTGAAACAAACAGCAACATGTCTAGCTTGTATCAGCTTCCAAACTTCTTTTCATTGAAGTTGGCCTCGTGCAATCTTCACAAGTTCCCTAATCTACAAGACCAATCCAATTTGATAAATCTAGACCTTTCAGACAACAAAATTGATGGAGAGATACCAAGCTGGATCTGGGAAGTTGGCAATGGAGGCCTTACGTATCTGAACCTTTCTCATAATCAATTAACAGGCCTCCAAGAGCCCTATGTTTTTCCGGATCTTGATGTCCTTGACATGCATTCCAATCGCCTCAGTGGGGAGCTTGTGGTTCCACCAGTAACTGCTTTTTGGATTGACTGTTCTGACAATCTTTTCAATTCATCACTCCCTGAAAGCATTGGTGTCAACCTCAGTAACGCATATTTCTTTTCGGTTTCTAACAATTTACTCAATGGAGAGATCCCTGAAACCATATGCAATGCTCCCTACCTAACGGTTCTTGATTTGTCAAACAATCATTTAACCGGAAGGATACCACGGTGTTTGATTGAATATGGTAGTGGCAGTATTGGTTTTGGGGTCCTGAACTTGGGAAACAATAGTCTCAGCGGTCGAATCGAGGGAACATTTCCGAGCTTCTGCGGTTTGAACACTTTAGACCTGCATGGAAACTTTTTGGAAGGGGAGATCCCAAAATCTCTTGTCAATTGCAGAATGCTACAAGTCTTAAACCTTGGCAACAACAGGATGATCGACACTTATCCGTGCTTTTTGCATAACAATACAAACTTGCGTGTACTTGTTCTGCGATCCAACAAGTTTCATGGCTCTGTGGGTTGTGGGGAAGACCAACAGAACAACTGGTCAAAGATTCAGATTGTGGACATAGCTCACAATAATTTTAATGGTACTGTCCCTGCACATTATTTCTGGCGATGGGATGCTATGATCACTCACAATCACAATGATGGAGAATGGGCAAGTAAGAAGCACCTAATTTTCATGGTGATGCAACTTAATAACTTGTACTATCAGGATACTGTGGCAGTCACTGTCAAAGGACGTGAGTTGGAGCTGCTGAAAATCTTGATAATCTTCACATCCATTGATATCTCAAGTAACAATTTTACAGGAGAAATACCAGACACAATTGGGAGACTCAACGCTCTGTATATCCTCAACATATCGCATAATGACTTCACAGGCCCTATCCCCTCATCTATCGGGAATTTGAGGCAGCTTGAATCATTGGACATGTCATCCAACAAACTAACTGGAGAGATCCCATTAGTGCTCACTGATCTTCCATTCCTTTCCGCCTTTGATCTGTCATACAATAAATTGGAAGGAAGAATCCCAACAGGCTCCCAGTTTCAGACTTTTGATGAGGATTCCTATGGAGGAAACAAGGGATTATGTGGGTTTCCTCTGAATAAAAGATGCAAAACTTCAGTGGCAGTATCATTACCACCAAAATCCCCAGAATCAAATGATGATGGGTATGATTGGCAGTCCCTATTCTATGGAATGGCAGCCGGGTCTGGATCATTAGCTGTTTTAGCGATCCTGTATTCCCTTTTCAAGAGACCTACAACCAGACAATGA

>LsVe4L

ATGAGAACCCAACTCTGGTTTTAGATTTTGTTGATAATTGGCTTTTACCTGATCTTGTTAGGGGTCAACTCCCCATCGGCATCTAGCCATTGTCAAACCGAGCAGCAATCGGTTTTGATCCGGTTGAAGAAAGAATTAGATTTCGATTCTTTGTTATCATCAAAATTAGTGTATTGGGAGCCAAATGCAGCAGATTGCTGCACCTGGACAGGTGTTACTTGCAGTGCTGGGGGTGTTGTCATCGGTCTAGATTTAAGCAACGAAACGATATCTGGTGGTATTGATGATTCTAGTTCTCTCTTCTGTTTAGAGAGTCTTGAGACGCTGAATCTGGCTGGAAATAACTTCAATTCCACGCCGATTCCTTCAGGATTTGGCAGTTTGACTAGTTTAAGGAATTTGAACTTGTCAAATTCGTGGTTTTCAGGCCAGATTCCTGGAGAATTGTCTCATCTGACAAAGCTTCAAGTTCTTGATCTGTCTTCTCTTTTCTCCTTTCACTCACTGAAACTTGAGAGCCCCAATCTAGCCATGCTTATCAAAAACCTGACACGGCTTAGAGTTGTTTATCTGGATGGTGTCAACATATCGGCACAAAAATCTGATTGGTGCCAGGCTTTGTCCTCATCTTTGCTTCGTTTGGAGGCTTTGAGCTTGTCAGATTGTCAACTTTCAGGCCCTTTAGATGAGTCTCTTGGTAAGCTACAGTCACTTTCTGTAATTCGACTGGCTAACAACAATCTTAATGCTTCGGTTCCAGATTTCTTTGGGAAGTTCAGGAATCTGACTACTCTGCATCTTGGTGCTTGTAATCTGCATGGAACATTTCCCACCAAGGTCCTCCAGTTGCAGCATTTACAGATTCTGGATTTATCTGTCAATAGGAATCTTCATGGTTCCTTACCAGGTTTTCCTGTGAACAGATCCCTTACAAGTTTAGTGCTTAGCAACACAAACCTTTCAGGGGAGATACCGGAATCTATCGGGAATCTTAAGAGCTTATCCCGGATAGAGCTTCCAAATTGCAATTTCAGAGGAAGAATTCCCAAATCCATGGAAAACCTCACCCAATTGACTTACCTTGACTTGTCCTCAAACAAGTTAACTGGCCAAATCCCATCCTTTCAGCTGTGCAAAAATCTTACCCACATTGACCTCTCAAGGAACAGTTTATCGGGTATCATCCCTTCTGCTCATTTCCAGGATCTTCAACATCTCATGTTAATTAATCTAAGGTTCAATACTTTCAACGGAAGCATACCTTCATCTCTGTTCAATCTGCAGCAATTGCAAAAGATACAGCTTTCCAACAACAATTTTGATGGCGTACTTACTGATTTTTCTAATCCATCAGCATCTTTATTGGACACTCTTGATTTAAGCAGTAACATGTTGAAAGGACAAATTCCCAAGTCTTTCTTTCAACTTGGAAGGCTTAATATCCTCTTGCTATCTTCAAACAACCTCAATGGCACGATTTATACAAAAGATTTCCAGGGTCTGAGCAACCTAACAGCCCTTGATCTTTCCTTCAACAATTTGTCAATTATCAACAGCCCAATCCCCTTGACACATCTTCCAAAGTTTTTCTCATTAAGGTTGGCTTCATGTAATCTTCATCATTTCCCTAAACTACAAAACCAATCAAGATTGATAAATCTAGACCTTTCAGACAACAAAATTGACGGAGAAATACCAAACTGGATCTGGCAAGTTGGCACCAGTTATGAAGGCCTTACATACATGAACCTTTCTCATAACCAGTTAACTTCCCTCCAAGAGCCCTATTTTCTGCCTGATCTTGGTGTCCTCGACCTGCATTCCAATGACCTCCATGGGGCGATTCCAATTCCACCAAAAACTGCAACCTTCATTGATTACTCCAACAATCTTTTCAATTCCTCACTCCCTGAAACCATTGGTGTAAACCTCACTTTTGCCTATTTCTTTTCGGTTTCCACAATTCACTCACTGGGGAGATCCCCGAAACCATATGCAATGCTCGCTACCTCAAAGTTGTTGATTTGTCAAACAATAATTTAACTGGAAGGATACCACGGTGTTTGATCGAATCTGGTAGTGGCAGTATTGGGGTACTCAACTTGGGTGGCAATCGTCTCAGCGGTCGAATCGAGGGAATATTCCCTAGCATCTGTGGTCTAAACACTCTAGACCTGCATGGAAACCGTTTAGAAGGGGAGATCCCAGGATCTCTTGTCAACTGCAACATGCTACAAGTCTTAAACCTTGGCAACAACAGGATGATCGACACTTATCCTTGCTCTTTGAGTAACAATATTACAAGTTTGCGTGTCCTTGTTCTCCGAAACAACAGGTTTCATGGGTCCATACATTGTGGTGAAGATCAACAGAACAAGTGGTCAAAGATTCAGATTCTGGACATAGCTCACAATAACTTTAATGGTACTGTCCCACCAGATTATTTCTGGAAATGGGATGCTATGAATATGATGACTCACAATCACACTGATGGAGAATGGGGAAGTAAGAAGCACATCAGTTTCAAGGTGATGCCACTTGATAACTTGTACTATCAGGATACTGTGGCAGTCACTGTCAAAGGACGTGAGTTGGAGCTGCTGAAAATTTTGACAATCTTCACATCCATTGATATCTCCAGCAACTGTTTTTCAGGAGAAATACCAGACACAATTGGGAGACTCACTGCTCTGTATATGCTCAACATATCACATAATGAATTCACAGGCTCTATCCCACCATCTATCGGGAATTTGAGTCAGCTTGAATCATTGGACATGTCTTCCAACAAACTAACTGGAAAGATCCCATCAGAGCTCACTGATCTTCCATTCCTTTCTGTTCTTAATCTATCATACAATCAATTAAAAGGAAGAATCCCAACAGGCTCCCAGTTTCAGACTTTTGATGGGGATTCCTATGGAGGAAACAAGGGATTATGTGGGTTGCCTCTGATTAGAAGTTGCACTACTTCAGTTATAATATCATCACCACCAAATTCCCAAGAGTCAAATGATGATGGGTATGATTGGCAATCCCTATTCTATGGAATGGCAGCCGGATCTGGATCATTAGCTGTTTTAGCGATCCTGTATTCCCTTTTCAAGAGACGTACAACCAGACAATGA

>LsVe1S

ATGAGAAACCACCTCTGTTTTCAGATTTTCTTCATACAGTTTTACTTAATTTTATTTGGTGTTTCTGGTATATGTCAAATCGAGCAGCAATCGATTTTGATCCGGTTGAAGAACGAATTACAGTTCAATTCTTCGTTATCATCAAAATTAGTGTCTTGGGACCCAAATGCAGCAGATTGCTGCACCTGGATAGGTGTTAATTGCAGCATTGGGGGTCAGGTTATCGGTCTAGATTTAAGCAACGAAACGATATCTGGTGGTATTGATGATTCTAGTTCTCTCTTCCGTTTAGAGAGTCTTGAGACGCTGAATCTGGCTGGAAATAACTTCAATTCCACGCCGATTCCTTCAGGATTTGGCAGTTTGACTAGTTTAAGGAATTTGAACTTGTCAAATTCGTGGTTTTCTGGCCAGATTCCTGGAGAATTGTCGCATCTGACAAAGCTTCAAGTTCTTGATCTGTCTTCTCTTTTCTCCTTTCGCTCACTGAAACTTGAGAGCCCCAATCTAGCCATGCTTATCAAAAACCTCACACAGCTTAAAGTTCTTCATCTGGATAGTGTGAACATATCGGCACAAAAATCAGATTGGTGCCAGGCTTTATCCTCTTCTTTGCTTGATTTGGAGGTTTTGAGTTTGTCAACTTGTCAACTTTCAGGCCCTTTAGATGGCTCCCTTGGGAACCTACACTCACTTTCTGTAATTCGTCTGGCTCAGAACAATCTTAGCACTCCAATTCCAGATTTCTTTGGGAATTTCAGAAATCTGACTATTCTGCATCTTGGTGCTTGTAATTTGCGTGGAACATTTCCCTCAAAGGTCCTGGAATTGCAGAAATTACAGAGTCTGGATCTTTCAAGCAATATGAATCTTCATGGTTCCTTATCAGATTTTCCTGTGAATGGATCCCTTCAAAGTTTAGTGCTCAGCAACACAAATCTTTCAGGAGCAATACCAGAATCTATCGGGAATCTTAAGAGCTTATCCCGGATAGAGCTTCCAAATAACAATTTCAGTGGAAGGATTCCCAAGTCAATGGAAAACCTCACCCAATTGACTTACCTTGACTTGTCCTCAAACAAGTTAACTGGCCAAATCCCATCCTTTCAGCTGTGCAAAAATCTTACCCACATTGACCTCTCAAGGAACAGTTTATCGGGTATCATCCCTTCTGCTCATTTCCAGGATCTTCAAAATCTTGTGTTAATTAATCTAAGGTTCAATACCTTCAATGGAAGCATACCTCCATCTCTGTTCAATCTGCAGCAATTGCAAAAGATACAGCTTTCCAACAACAATTTTGATGGCGTACTTACTGATTTTTTAAATGCATCTGCATCTTTATTGGACACTCTTGATTTAAGCAGTAACAAGTTGAAAGGACAAATTCCCAAGTCTTTCTTTCAACTTGGAAGACTTAATATCCTCTTGCTATCGTCCAACAACCTCAATGGCACGATCCACACAAGTGAATTCCAGGGTCTGAGCAACCTAACAACCCTTGATCTTTCCTTCAACAACTTGTCGATTATCACAAGCCCAATCCCCTTGCCTCGTCTTCCAAAGTTTTTCTCATTAAAGTTGGCTTCATGCAATCTTCAACATTTCCCTAAACTACAAAACCAATCAAGATTGATAAATCTAGACCTTTCAGACAACAAAATTGATGAAGAAATACCAAACTGGATCTGGGAAGTTCGCAGTGAAGGCCTTGCATACATGAACCTTTCTCATAACCAGTTAACTGGTCTCCAAGAGCCCTATGTTCTTCCTGATCTTGCTGTCCTCGACCTGCATTCCAATCGCCTCCACGGGGCGATTCCAATTCCACCACAAACTGCGACCTTCATTGATTACTCCAACAATCGTTTCAATTCCTCACTCCCTGAAACCATTGGCATAAACCTCTTGTATGCATATTTCTTTTCGGTTTCTAACAATTCACTCTCTGGGGAGATTCCCGAATCCATCTGCGATGCTACCTACCTCAAGGTTCTTGATTTATCAAACAATCTGTTCACTGGAAGGATACCACAGTGTTTAATTGATTCCGGTGGAAGTCTTGGGGTGCTCAACTTGGGAAACAATAATCTTACTGGTCGAATCGAGGGAATATTCCCTACCACCTGTGGTCTAAACACTCTAGACCTTCATAGCAACTCTTTGGAGGGGGAGATCCCACGTTCTCTTGCCAATTGCACGATGCTAGAAGTCTTAAACCTTGGCAACAACAAGATGAACGACACGTATCCTTGCTCTTTGCGTAACAATACGAATCTGCGTGTCCTTGTTCTGCGGAACAACAAGTTTCATGGGTCCGTGCGTTGTAGTGAACAACAACGCAACAACTGGTCAAATATTCAGATTGTAGACATAGCTCACAATAGCTTCAATGGTCCTGTTCCTGCAGATTGTTTCTGGCAATGGGATGCTATGATCAGTAATGACGATGGAGAAGCATCAGGTAAGAAACACCTAAGTTTCACAGTCTTATCTCTTGATCCGATCTACTATCAGGATACTGTGGAAGTCACAATCAAAGGACTTGAGCTGGAGCTGGTGAAAATCTTGACAATCTTCACATCCATTGACATCTCAAGTAACCGTTTTTCTGGTGAAATACCTGACACAATTGGGAGACTCAATGCTCTGTATATGTTCAATGTATCATATAATGAGTTCACAGGCCCTATCCCCTCATCTATCGGGAATTTGAGGCAGCTTGAATCATTAGACATGTCATCAAACAATCTAACTGGAAATATCCCATCAGAGCTCACTGCTCTTCCATTCCTTTCTGTTCTTAATCTATCATACAATCAATTGGAAGGAAGAATCCCAACAGGCTCCCAGTTTCAGACCTTCCAAAACACATCATACAAAGGAAATATAGGATTATGTGGGTCTCCATTGAACAAAATATGCACCACTTCAGATGTAATCAAACCAAAGCACACAACAACAGTTTTTGAAGATTCTAATGGATATGATTGGCAGTTTATATTCACTGGAGTGGGATTCGGGGCAGGTGCAGCCATTGTTGTAGGGCCTCTCGTGTTGTCTAAGCAAGGGAGAAACTTCTGGGACAAGTACACAAACAAGATTGTGGAGATTATCTGTCTGGTTTTGGGTATCCATTATGCAGCTTATGGGTTATTCAACCAAGATGAACATGATGAAAATGAGACACAAGACATGGATGAAAGCTCAGATGAAGATGAATTTCTGTCAGAAGTTGATCAGTCTAAAGGTAGGTATTGTGTGTTTTGTACCAAACTTGATTTCTCTAGGAAACAAGCCATTCATGATACAAAATGCACTTGCTTTGACCAAACACCAACATTGTCTACTTCAGTGTCCACTTCTTCAACGGAAGTAGAGTCTCCATTTACCAAATTGTGA

>LsVe2S

ATGAGAAGCCAACTATGGTTTCAGATTTTGTTGATAATTGGCTTTTACGTGATCTTTTTTGGGGTCACTTCCAAACTAGTTTCTGGCCATTGTCAAAACGAGCAGCAATCGGTTTTGATCCGGTTGAAGAAAGAATTAGATTTCGATTCTTTGTTATCATCAAAATTAGTGTCTTGGGACCCAAATGCAGCAGATTGCTGCACCTGGATAGGTGTTACTTGCAGTGCTGGGGGTGTTGTCATCGGTCTAGATTTAAGCAACGAGACGATATCTGATGGTATTGATAATTCTAGTGCTCTCTTCCGTTTAGAGAATCTTGAGACGCTGAATCTGGCTGAAAATAACTTCAATTCCACACCGATTCCTTCAGGGTTTGGCAGTTTGACTAGTTTGAGGAATTTGAACTTGTCCAATTCTGGTTTTTCAGGGCAGATTCCTGGTGAATTGTCTCATCTGACAAAGCTTCAAGTTCTTGATCTGTCTTCTCTGTTCTCCTTTCGCTCACTGAAACTTGAGAGCCCCAATCTAGCCACGCTTATCAAAAACCTCACACAGCTTAAAGTTCTTCATCTGGATAGTGTGAACATATCGGCACAAAAATCAGATTGGTGCCAGGCTTTATCCTCTTCTTTGCTTGATTTGGAGGTTTTGAGTTTGTCAACTTGTCAACTTTCAGGCCCTTTAGATGACTCCCTTGGGAACCTACAGTCACTTTCTGTAATTCGTCTGGCTCAGAACAATCTTAGCACTCCAATTCCAGATTTCTTTGGAAATTTCAGAAATCTGACTATTCTGCATCTTGGTGCTTGTAATTTGCGTGGAACGTTTCCCTCAAAGGTCCTGGAATTGCAGAAATTACAGAGTCTGGATCTTTCAAGCAATATGAATCTTCATGGTTCCTTATCAGATTTTCCTGTGAATGGATCTCTTCAAAGTTTAGTGCTCAGCAACACAAACCTTTCAGGAGCAATACCAGAATCTATCGGGTATCTTAAGAGCTTATCCCGGATTGAGCTTCCAAATAACAATTTCAGTGGAAGAATTCCCAAGTCAATGGAAAACCTCACCCAATTGACTTACCTTGACTTGTCCTCAAACAAGTTAACTGGCCAAATCCCATCCTTTCAGCTGTGCAAAAATCTTACCCACATTGACCTCTCAAGGAACAGTTTATTGGGTATCATCCCTTCTGCTCATTTCCAGGATCTTCAAAATCTTGTGTTAATTAATCTAAGGTTCAATACCTTCAACGGAAGCATACCTTCATCTCTGTTCAATCTGCAGCAATTGCAAAAGATACAGCTTTCCAACAACAATTTTGATGGCGTACTTACTGATTTTTTAAATGCATCTGCATCTTTATTGGACACTCTTGATTTAAGCAGTAACAAGTTGAAAGGACAAATTCCCAAGTCTTTCTTTCAACTTGGAAGGCTTAATATCCTCTTGCTATCTTCAAACAACCTCAATGGCATGATTTATACAAAAGATTTCCAGGGCCTAAGCAACCTAACAACCCTTGATCTTTCCTTCAACAACTTGTCAGTTATCAACAGCCCAATCCCTTTGCCTTATCTTCCAAAGTTTTTCTCATTAAAGTTGGCTTCATGCAATCTTCAGCATTTCCCTAAACTACACAACCAATCAAGATTGATAACTCTAGACCTTTCAGACAACAAAATTGATGGAGAAATACCAAACTGGATCTGGCAAGTTGGCACCAGTTATGGAGGCCTTTCGTATCTGAACCTTTCTCGTAACCAGTTAAATAGCATCCAAGAGCCCTATTTTGTGCCCGATCTTGCTGTCCTCGACCTGCATTCCAATCGCCTCCATGGGGCGATTCCAATTCCACCACAAATTGCAACCTTCATTGATTACTCCAACAATCGTTTCAATTCCTCACTCCCTGAAACCATTGGTGTAAACCTCACTATTGCCTCTTTCTTTTCGGTTTCTAACAATTCACTCTCTGGGGAGATTCCCGAATCCATCTGCAACGCTACATACCTCAAGGTTCTGGATTTGTCAAACAACAATTTAACTGGAAGTATACCACCGTGTTTGATCGAATCTGGTGGTGGCAGTATTGGGGTACTCAACTTGGGAAGCAATAGTCTCAGTGGAAGAATCGAGGGAATATTCCCAAGCACATGTGGCCTAAACACTTTAGACCTTCATGGAAACCATTTGGAAGGGGAGATCCCACGTTCTCTTGCCAACTGCACAATGCTAGAAGTCTTAAACCTTGGCAACAACAGGATGATCGACACTTATCCTTGCTCTTTGAGTAACAATATTACAAGTTTGCGTGTCCTTGTTCTCCGAAACAACAGGTTTCATGGGTCCATACATTGTGGTGAAGATCAACAGAACAAGTGGTCAAAGATTCAGATTCTGGACATAGCTCACAATAACTTTAATGGTACTGTCCCACCAGATTATTTCTGGAAATGGGATGCTATGAATATGATGACTCGCAATCACAATGATGGAGAATCGGTAGGTAAGAAGCACATCAGTTTCAAGGTGATGCCACTTGATAACTTGTACTATCAGGATACTGTGGCAGTCACTGTCAAAGGACGTGAGTTGGAGCTTGTGAAAATCTTGACAATCTTCACATCCATCGACATCTCCAGCAATCGATTTTCAGGAGAAATACCGGACACAATTGGGAAACTCAATGCTCTGTATATGCTCAACATATCACATAATGACTTCACAGGTCCTATCCCCTCATCTTTCGGGAGTTTGAGGCAGCTTGAATCATTGGACATGTCATCCAACAAACTAACTGGAGAGATCCCATTAGTGCTCACTGATCTTCCATTCCTTTCCGCCTTTGATCTGTCACACAATAAATTGGAAGGAAGAATCCCAACAGGCTCCCAGTTTCAGACTTTTGATGAGGATTCCTATGGAGGAAACAAGGGATTATGTGGGTTTCCTCTGAATAAAAGTTGTAAGACTTCAGTTGCAGTATCATTACCACCAAAATCCCGAGAATCAAATGATGGGTATGATTGGCAGTCCCTATTCTATGGAATGGCAGCCGGGTCTGGATCATTAGCTGTTTTAGCGATCCTGTATTCCCTTTTCAAGAGACCTACAACCAGACAATGA

>LsVe3S

ATGAGAAGCCAACTCTGGTTTCAGATTTTGTTGATAATTGGCTTTTACCTGATCTTGTTAGGGGTCAACTCCCCATCGGCATCTAGCCATTGTCAAACCGAGCAGCAATCGGTTTTGATCCGGTTGAAGAAAGAATTACATTTCGATTCTTTGTTATCATCAAAATTAGTGTATTGGGAGCCAAATGCAGCAGATTGCTGCACCTGGACAGGTGTTACTTGCAGTGCTGGGGGTGTTGTCATCGGTCTAGATTTAAGCAACGAAACGATATCTGGTGGTATTGATGATTCTAGTTCTCTCTTCCGTTTAGAGAGTCTTGAGACGCTGAATCTGGCTGGAAATAACTTCAATTCCACGCCGATTCCTTCAGGATTTGGCAGTTTGACTAGTTTAAGGAATTTGAACTTGTCAAATTCGTGGTTTTCAGGCCAGATTCCTGGAGAATTGTCTCATCTGACAAAGCTTCAAGTTCTTGATCTGTCTTCTCTTTTCTCCTTTCACTCACTGAAACTTGAGAGCCCCAATCTAGCCATGCTTATCAAAAACCTGACACGGCTTAGAGTTGTTTATCTGGATGGTGTCAACATATCGGCACAAAAATCTGATTGGTGCCAGGCTTTGTCCTCATCTTTGCTTCGTTTGGAGGCTTTGAGCTTGTCAGATTGTCAACTTTCAGGCCCTTTAGATGAGTCTCTTGGTAAGCTACAGTCACTTTCTGTAATTCGTCTGGCTCTGAACAATCTTAATGCTTCAGTTCCAGATTTCTTTGGGAAGTTCAGGAATCTGACGGCTCTGCATCTTGGTAATTGTAATCTGCATGGAACATTTCCCACCAAGGTCCTCCAGTTGCAGCGTTTACAGATTCTGGATTTATCTGTCAATAGGAATCTTCATGGTTCCTTACCAGGTTTTCCTGTGAACAGATCCCTTAGAAGTTTAGTGCTTAGCAACACAAACCTTTCAGGGGAGATACCGGAATCTATTGGGAATCTTAAGAGCTTATCCCGGATAGAGCTTCCAAATTGCAATTTCAGAGGAAGAATTCCCAAATCCATGGAAAACCTCACCCAATTGACTTACCTTGACTTGTCCTCAAACAAGTTAACTGGCCAAATCCCATCCTTTCAGCTGTGCAAAAATCTTACCCACATTGACCTCTCAAGGAACAGTTTATCGGGTATCATCCCTTCTGCTCATTTCCAGGATCTTCAACATCTCATGTTAATTAATCTAAGGTTCAATACTTTCAACGGAAGCATACCTTCATCTCTGTTCAATCTGCAGCAATTGCAAAAGATACAGCTTTCCAACAACAATTTTGATGGCGTACTTACTGATTTTTCTAATCCATCAGCATCTTTATTGGACACTCTTGATTTAAGCAGTAACATGTTGAAAGGACAAATTCCCAAGTCTTTCTTTCAACTTGGAAGGCTTAATATCCTCTTGCTATCTTCAAACAACCTCAATGGCACGATTTATACAAAAGATTTCCAGGGTCTGAGCAACCTAACAGCCCTTGATCTTTCCTTCAACAATTTGTCAATTATCAACAGCCCAATCCCCTTGACACATCTTCCAAAGTTTTTCTCATTAAGGTTGGCTTCATGTAATCTTCATCATTTCCCTAAACTACAAAACCAATCAAGATTGATAAATCTAGACCTTTCAGACAACAAAATTGATGGAGAAATACCAAACTGGATCTGGCAAGTTGGCACCAGTTATGAAGGCCTTACATACATGAACCTTTCTCATAACCAGTTAACTTCCCTCCAAGAGCCCTATTTTCTGCCTGATCTTGGTGTCCTCGACCTGCATTCCAATGACCTCCATGGGGCGATTCCAATTCCACCAAAAACTGCAACCTTCATTGATTACTCCAACAATCTTTTCAATTCCTCACTCCCTGAAACCATTGGTGTAAACCTCACTTTTGCCTATTTCTTTTCGGTTTCCAACAATTCACTCACTGGGGAGATCCCCGAAACCATATGCAATGCTCGCTACCTCAAAGTTGTTGATTTGTCAAACAATAATTTAACTGGAAGGATACCACGGTGTTTGATCGAATCTGGTAGTGGCAGTATTGGGGTACTCAACTTGGGTGGCAATCGTCTCAGCGGTCGAATCGAGGGAATATTCCCTAGCAACTGTGGTCTAAACACTCTAGACCTGCATGGAAACCGTTTAGAAGGGGAGATCCCAGGATCTCTTGTCAACTGCAACATGCTACAAGTCTTAAACCTTGGCAACAACAGGATGATCGACACTTATCCTTGCTCTTTGAGTAACAATATTACAAGTTTGCGTGTCCTTGTTCTCCGAAACAACAGGTTTCATGGGTCCATACATTGTGGTGAAGATCAACAGAACAAGTGGTCAAAGATTCAGATTCTGGACATAGCTCACAATAACTTTAATGGTACTGTCCCACCAGATTATTTCTGGAAATGGGATGCTATGAATAACAATCACACTGATGGAGAATGGGGAAGTAAGAAGCACATCAGTTTCAAGGTGATGCCACTTGATAACTTGTACTATCAGGATACTGTGGCAGTCACTGTCAAAGGACGTGAGTTGGAGCTGGTGAAAATTTTGACAATCTTCACATCCATTGATATCTCCAGCAACCGTTTTTCAGGAGAAATACCAGACACAATTGGGAGACTCACTGCTCTGTATATGCTCAACATATCACATAATGAATTCACAGGCTCTATCCCACCATCTATCGGGAATTTGAGTCAGCTTGAATCATTGGACATGTCTTCCAACAAACTAACTGGAAAGATCCCATCAGAGCTCACTGATCTTCCATTCCTTTCTGTTCTTAATCTATCATACAATCAATTAAAAGGAAGAATCCCAACAGGCTCCCAGTTTCAGACTTTTGATGGGGATTCCTATGGAGGAAACAAGGGATTATGTGGGTTGCCTCTGATTAGAAGTTGCACTACTTCAGTTATAATATCATCACCACCAAATTCCCAAGAGTCAAATGATGATGGGTATGATTGGCAATCCCTATTCTATGGAATGGCAGCCGGATCTGGATCATTAGCTGTTTTAGCGATCCTGTATTCCCTTTTCAAGAGACGTACAACCAGACAATGA
